# SOX9-induced Generation of Functional Astrocytes Supporting Neuronal Maturation in an All-human System

**DOI:** 10.1007/s12015-021-10179-x

**Published:** 2021-05-12

**Authors:** Katrien Neyrinck, Johanna Van Den Daele, Tim Vervliet, Jonathan De Smedt, Keimpe Wierda, Melissa Nijs, Tom Vanbokhoven, Astrid D’hondt, Mélanie Planque, Sarah-Maria Fendt, Pei-Yu Shih, Frederik Seibt, Juan Pita Almenar, Mohamed Kreir, Devesh Kumar, Vania Broccoli, Geert Bultynck, Andreas Ebneth, Alfredo Cabrera-Socorro, Catherine Verfaillie

**Affiliations:** 1 Stem Cell Institute, Department of Development and Regeneration, KU Leuven, Leuven, 3000 Belgium; 2 Laboratory of Molecular and Cellular Signalling, Department of Cellular and Molecular Medicine, KU Leuven, Leuven, Belgium; 3 Electrophysiology Expert Unit, VIB-KU Leuven Center for Brain & Disease Research, Leuven, 3000 Belgium; 4 Laboratory of Cellular Metabolism and Metabolic Regulation, Department of Oncoloy, KU Leuven and Leuven Cancer Institute (LKI), Leuven, 3000 Belgium; 5 Laboratory of Cellular Metabolism and Metabolic Regulation, VIB-KU Leuven Center for Cancer Biology, VIB, Leuven, 3000 Belgium; 6 Division of Janssen Pharmaceutica, Janssen Research & Development, Beerse, 2340 Belgium; 7 Division of Neuroscience, IRCCS, San Raffaele Scientific Hospital, 20132 Milan, Italy; 8 Institute of Neuroscience, National Research Council (CNR), 20129 Milan, Italy

**Keywords:** Astrocytes, Pluripotent stem cells, Differentiation protocol, Genome engineering, All-human co-culture system

## Abstract

Astrocytes, the main supportive cell type of the brain, show functional impairments upon ageing and in a broad spectrum of neurological disorders. Limited access to human astroglia for pre-clinical studies has been a major bottleneck delaying our understanding of their role in brain health and disease. We demonstrate here that functionally mature human astrocytes can be generated by *SOX9* overexpression for 6 days in pluripotent stem cell (PSC)-derived neural progenitor cells. Inducible (i)SOX9-astrocytes display functional properties comparable to primary human astrocytes comprising glutamate uptake, induced calcium responses and cytokine/growth factor secretion. Importantly, electrophysiological properties of iNGN2-neurons co-cultured with iSOX9-astrocytes are indistinguishable from gold-standard murine primary cultures. The high yield, fast timing and the possibility to cryopreserve iSOX9-astrocytes without losing functional properties makes them suitable for scaled-up production for high-throughput analyses. Our findings represent a step forward to an all-human iPSC-derived neural model for drug development in neuroscience and towards the reduction of animal use in biomedical research.

**Graphical Abstract:**

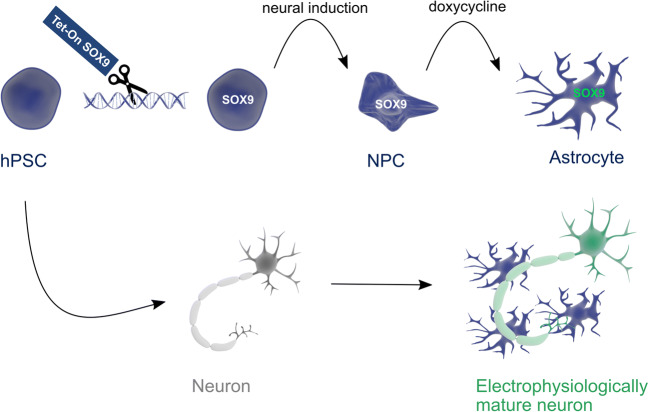

**Supplementary Information:**

The online version contains supplementary material available at 10.1007/s12015-021-10179-x.

## Introduction

Astrocytes are the most abundant cell type in the brain and are crucial for healthy central nervous system functioning [1]. Dysfunction of astrocytes has been identified to play a key role in brain-ageing and neurodegenerative diseases, including Alzheimer’s disease (AD) and amyotrophic lateral sclerosis (ALS) [2, 3]. *In vivo*, astrocytes provide trophic and metabolic neuronal support [4, 5] as well as maintaining proper synapse and blood-brain-barrier (BBB) functioning [6, 7]. In addition, astrocytes take up excessive extracellular ions and neurotransmitters preventing neuronal toxicity [8, 9]. These functions of astrocytes are also key for *in vitro* maturation of neurons. Especially *in vitro* cultured neurons derived from pluripotent stem cells (PSCs) need astrocytic support to acquire typical neuronal electrophysiological properties so they become amenable for functional studies [10–13]. To achieve PSC-derived neuronal maturation, most studies have used primary rodent-derived astrocytes. However, rodent astrocyte functions are not fully conserved in higher order mammals. In human or primate brains for instance, more elaborate neuronal networks require greater numbers and more complex astrocytes [7, 14]. Therefore, human PSC-derived astrocytes would preferably be used. Initially, PSC-derived astrocytes were generated by mimicking developmental astroglial differentiation using only growth factors and/or small molecules [15–18]. However, such protocols are very labor intensive and lengthy, ranging from 75 to 600 days. To bypass this time-consuming process, transcription factor (TF) overexpression can be used to speed up the differentiation process, as has been done for neurons (*NGN2* overexpression), for example [10]. In 2015, Caiazzo et al. found that overexpression of the TFs *NFI-A, NFI-B* and *SOX9* was sufficient to convert mouse fibroblasts into astrocyte-like cells [19]. Three recent studies have used this approach to create human astrocytes from PSCs, by inducing expression of either *NFI-A* and *SOX9* or *NFI-B* and *SOX9* in PSCs, or *NFI-A* as the sole TF in PSC-derived neural progenitor cells (NPCs) [20–22]. During mouse development, *SOX9* is already expressed at E10.5 and binds to astrocyte (and oligodendrocyte) specific genes, such as NFI-A that becomes expressed at E11.5 [23, 24]. Many SOX9 targeted regions also contain NFI-motifs, to which NFI-A binds. SOX9 and NFI-A synergistically activate gene expression during the gliogenic switch [24]. During further astrocyte (but not oligodendrocyte) maturation steps, SOX9 and NFI-A remain expressed activating mature astrocytic genes, with a crucial role of SOX9 as its loss prevents astrogenesis [25, 26]. Because of the central role of SOX9 in these developmental steps and based on our previous findings that nearly 100 % pure oligodendrocytes can be generated from PSCs when *SOX10* (without co-factors) is overexpressed in NPCs [27, 28], we here hypothesized that induced expression of *SOX9* without the need for the co-factors, *NFI-A* or *NFI-B*, might suffice to create astrocytes.

We demonstrated that *SOX9* overexpression by doxycycline in iPSC-derived NPCs is indeed sufficient to generate a stable astrocyte phenotype already from 6 days after doxycycline treatment and remains stable for at least 4 weeks following removal of doxycyline. Transcriptome analysis indicated that the induced (i)SOX9-astrocytes attain a maturation state intermediate between freshly isolated pre- and post-natal human astrocytes. iSOX9-astrocytes displayed robust glutamate uptake, calcium responses towards ATP and acetylcholine (Ach) and secreted cytokines/growth factors after stimulation with TNF-α and/or IL-1β. Most importantly, we demonstrate that iSOX9-astrocytes support synchronous activity of iNGN2-PSC neuronal progeny to levels higher than seen in gold-standard rat neuronal primary cultures. The high yield (> 100 astrocytes per PSC), the possibility of cryopreservation and fast timing (± 40 days) of the protocol makes iSOX9-astrocytes a useful cell source for scaled up-production for high-throughput analyses of human iPSC-derived neural models and drug development, while sustaining the 3R principle related to animal use in biomedical research.

## Materials and Methods

### Cell Culture and Genome-engineering

 Normal donor iPSCs (SIGi001-A, purchased from Sigma) were cultured at 37°C and 5 % CO_2_ on matrigel-coated 6-well plates (Cornig) with E8 flex medium (Gibco) and passaged twice a week using 0.5 mM EDTA (Gibco). First, the *AAVS1* recombination master cell line was created as decribed before [29]. Briefly, iPSCs were detached using accutase (Sigma) and single cell suspensions (10^6^ cells) were resuspended in 100 µl Nucleofector Solution™ (Lonza) with 5 µg of a donor plasmid and 2 µg mRNA coding for the left and right ZFNs. The donor plasmid contains homology arms flanking the ZFN-mediated double strand break with in between the homology arms a combination of GFP and hygromycin/thymidine kinase (TK) located between FRT and FRT3 sites. Following nucleofection with the Amaxa nucleofector device (program F16), single cells were plated on matrigel-coated plates in mTeSR^TM^1 medium (Stemcell Technologies) supplemented with rock inhibitor (Y-27632, VWR). When small colonies appeared, hygromycin (Hygromycin B, Thermoscientific) selection was performed by gradually increasing hygromycin concentrations from 50 to 300 µg/ml. Single colonies were manually picked and cultured separately. Quality control consisted of 5’ junction PCR to assure insertion inside the *AAVS1* locus and southern blot to detect possible random integration events.

iPSC clones that passed all QC, were subsequently subjected to recombinase-mediated cassette exchange (RMCE) to replace the GFP-Hygro-TK cassette with a cassette containing FRT and FRT3 sites in identical orientation, a promotorless puromycin cassette for gene trapping and an inducible TET-ON system for overexpression of *SOX9* (SIGi001-A-20 cells). Nucleoporation and subsequent selection was again performed as described by Ordovas et al. [29]. Correctly recombined cells were selected by initial positive selection using puromycin (Sigma; 120 to 300 ng/µl) followed by negative selection with 0.5 µM Fialuridine (FIAU, Sigma).

iCell astrocytes were commercially bought from Cellular Dynamics and are human iPSC-derived astrocytes. fHA were commercially bought from ScienCell and were derived from fetal cortex. Both commercially bought astocytes were cultured in DMEMF12 Glutamax™ (Gibco) supplemented with G5 (Gibco) and HB-EGF (Peprotech; 10 ng/mL).

### Generation of Lentiviral Particles

The plasmids needed for the generation of lentiviral vector particles are psPAX2 (Addgene #12260), pMD2.G (Addgene #12259), FUW-M2rtTA (Addgene #20342) and the plasmids encoding *SOX9*, *NFI-A* and *NFI-B* under a TET-ON promotor were kindly provided by prof. Broccoli from the San Raffaele Scientific Institute in Milan. Lenti-X HEK293T cells (ClonTech) were plated at a density of 125 000 cells/cm^2^ in HEK medium consisting of DMEM high glucose Glutamax™ (Gibco) supplemented with 10 % FBS (ThermoFisher), 100 U/ml Penicillin-Streptomycin (Gibco) and 1X sodium pyruvate (Gibco). The next day when cells reached 90 % confluency, they were transduced with 4 µg pMD2.G, 12 µg psPAX2 and 12 µg plasmid encoding for either *M2rtTA*, *SOX9*, *NFI*-*A* or *NFI-B* with the use of FuGENE® HD Transfection Reagent (Promega). After six hours, medium was changed to regular HEK medium and lentiviral vector particles were collected, filtered using a 0.45 μm Steriflip (Millipore) and stored at -80 °C 36 h later.

### Lentiviral Screen for Astrocyte Generation

Single cell suspensions of iPSCs were plated at 150 000 cells/cm^2^ in matrigel-coated plates in mTeSR^TM^1 medium supplemented with 1X Revitacell (Gibco). Medium was changed the next day to neural induction (NI) medium consisting of NMM supplemented with 1 µM LDN-193,189 (Miltenyi) and 10 µM SB431542 (Tocris). NMM consists of a 1:1 mixture of Neurobasal medium (Gibco) and DMEMF12 Glutamax™ supplemented with 0.5X Glutamax™ (Gibco), 50 U/ml Penicillin-Streptomycin, 0.5X B27 (Gibco), 0.5X N2 (Gibco), 0.5X MEM-NEAA (Gibco), 0.5X sodium pyruvate, 0.025 % human insulin (Sigma) and 50 µM 2-mercaptoethonal (Gibco). NI medium was changed daily for 12 consecutive days. Afterwards, cells were detached using accutase and plated single cell at a density of 65 000 cells/cm^2^ in NMM with 20 ng/ml bFGF (Peprotech) and 1X Revitacell. The next day, different combinations of the TF-containing lentiviral vectors (*NFI-A, NFI-B, SOX9, NFI-A + SOX9, NFI-B + SOX9, NFI-A + NFI-B + SOX9*) combined with an m2rtTA lentiviral vector were added to the wells. After 1 day, medium was changed to DMEMF12 Glutamax™ supplemented with 1X N2 and 3 µg/ml doxycycline (Doxycycline hyclate, Sigma). Medium was changed every other day for 7 days.

### iSOX9-astrocyte Differentiation

Single cell suspensions of SIGi001-A-20 iPSCs were plated at density of 150 000 cells/cm^2^ on matrigel-coated plates in mTeSR^TM^1 medium supplemented with 1X Revitacell. The next day, medium was changed to NI medium with daily medium changes for 12 consecutive days. On day 12, cells were detached using accutase and single cell suspension was plated on matrigel coated plates at 65 000 cells/cm^2^ in NMM supplemented with 20 ng/ml bFGF and 1X Revitacell. The following day, medium was replaced to maturation medium. Maturation medium consists of a 1:1 mixture of Neurobasal medium and DMEMF12 Glutamax™ supplemented with 1X N2, 1X sodium pyruvate, 1X Glutamax™, 0.5 mM N-acetyl-cysteine (Sigma), 0.1 mM dbcAMP (Sigma), 10 ng/ml CNTF (Peprotech), 10 ng/ml BMP4 (Peprotech) and 5 ng/ml HB-EGF (Peprotech). For the initial 6 days, maturation medium was supplemented with 3 µg/ml doxycycline and replaced every other day. From then onwards, cells were maintained in maturation medium without doxycycline and medium was changed twice a week. When the cells were > 90 % confluent, they were harvested with accutase and replated at 20 000 cells/cm^2^ in maturation medium.

### RT-qPCR

RNA was isolated with the Quick-RNA Microprep Kit (Zymo research) according to the manufacturer’s instructions. cDNA was made starting from 500 ng RNA using the SuperScript™ III First-Strand Synthesis System (Thermofisher) kit, followed by RT-qPCR using Platinum SYBR Green qPCR Supermix-UDG (Thermofisher). Primer sequences can be found in Table 1.

**Table 1:** RT-qPCR primer sequences

Gene	Forward primer	Reverse primer
*OCT4*	GATGGCGTACTGTGGGCCC	TGGGACTCCTCCGGGTTTTG
*PAX6*	AGGCCCTGGAGAAAGAGTTTG	TTTGGCTGCTAGTCTTTCTCG
*NESTIN*	TCAGCTTTCAGGACCCCAAG	TGGGAGCAAAGATCCAAGACG
*TUBB3*	CCTCCGTGTAGTGACCCTT	GGCCTTTGGACATCTCTTCAG
*SOX9*	CGTTCTTCACCGACTTCCTC	CTGGGCAAGCTCTGGAG
*NFI-A*	CTTCATGCCATCCACTTGACT	CTTTACCCAGCACATCCTCTAC
*NFI-B*	GATCATTGTGGCTTGGACTTC	GCCACATCATATCACAGTATCAGT
*CD44*	GAGATGCTGTAGCGACCATT	GACACCATGGACAAGTTTTGG
*S100B*	TGGCCCTCATCGACGTTTTC	ATGTTCAAAGAACTCGTGGCA
*GFAP*	GTCCCCCACCTAGTTTGCAG	TAGTCGTTGGCTTCGTGCTT
*ALDH1L1*	TCCAGACCTTCCGCTACTTTG	CAGGGGATAGTTCCAGGGGAT

### Flow Cytometry

Cells were detached using accutase and fixed for 15’ at RT in 4 % PFA (Sigma). Cells were washed with PBS and permeabilized with 100 % methanol (VWR) for 30’ at 4˚C. After washing with PBS, cells were incubated with primary antibodies for 45’ at 4˚C, washed and incubated with secondary antibodies for 30’ at 4˚C. After washing with PBS, flow cytometry was performed using the FACSCanto HTS device (BD Biosciences) acquiring 10,000 cells and data analyzed using FACSDiva software (version 6.1.2). Note that all centrifugation steps were done for 5’ at 600 rcf. List of antibodies can be found in Table 2. As control, samples only stained with secondary antibodies were used.

**Table 2:** List of antibodies used

	Antibody	Dilution	Company
Primary antibodies	Rabbit anti-S100B	1/100 (Flow cytometry) 1/200 (IF)	Abcam
	Rabbit anti-EAAT1	1/1000 (Flow cytometry) 1/200 (IF)	Abcam
	Rat anti-GFAP	1/1000 (IF)	Thermo Fisher Scientific
	Mouse anti-ALDH1L1	1/500 (IF)	Abcam
Secondary antibodies	Goat anti-rabbit Alexa488	1/500	Thermo Fisher Scientific
	Goat anti-rat Alexa555	1/500	Thermo Fisher Scientific
	Goat anti-mouse Alexa555	1/500	Thermo Fisher Scientific

### Immunostaining and Fluorescence Microscopy

Cells were fixed with 4 % PFA for 15’ at RT. After washing with PBS, cells were blocked and permeabilized for 20’ at RT with 5 % goat serum (Dako) and 0.1 % Triton X-100 (Sigma), all diluted in PBS. Next, primary antibodies were diluted in 5 % goat serum and added to the cells at 4 °C. The next day, cells were washed with PBS, and secondary antibodies diluted in Dako REAL Antibody Diluent (Dako) were added to the cells for 45’ at RT. Afterwards, the cytoplasm was stained using Cellmask (Thermo Fisher Scientific, diluted 1/2500 in PBS) for 30’ followed by a nuclear staining with Hoechst33342 (Sigma, diluted 1/1000 in Dako REAL Antibody diluent) for 10’, both at RT. The list of antibodies can be found in Table 2. Images were acquired using the Operetta High Content Imaging System (PerkinElmer). Consecutive image analysis was performed using automatic and unbiased object segmentation and counting in each channel using the image analysis platform Columbus (PerkinElmer). In short, nuclei (Hoechst staining) was the first sub-cellular structure to be segmented and used as a reference point for cytoplasm segmentation (Cellmask staining). Both segmented cell regions were selected for each separate immunofluorescence experiment and each time between 2500 and 14,000 cells were considered. Within the selected cell population, the mean fluorescence intensity was used to set a threshold for calculating the percentage of positive cells for each marker. In addition, the integrated density was calculated by dividing the mean fluorescence intensity by the area of the cytoplasm (in µm^2^).

### RNA Sequencing

#### RNA Quality Control

RNA concentration and purity were determined spectrophotometrically using the Nanodrop ND-1000 (Nanodrop Technologies) and RNA integrity was assessed using a Bioanalyser 2100 (Agilent) and a 5300 Fragment Analyzer (Agilent).

#### Library Preparation

Per sample, an amount of 100 ng of total RNA was used as input. For samples which contained less than 100ng, all RNA was used. Using the Illumina TruSeq® Stranded mRNA Sample Prep Kit (protocol version: Part # 1000000040600 v00 October 2017), poly-A containing mRNA molecules were purified from the total RNA input using poly-T oligo-attached magnetic beads. In a reverse transcription reaction using random primers, RNA was converted into first strand cDNA and subsequently converted into double-stranded cDNA in a second strand cDNA synthesis reaction using DNA Polymerase I and RNAse H. The cDNA fragments were extended with a single ‘A’ base to the 3’ ends of the blunt-ended cDNA fragments after which multiple indexing adapters were ligated introducing different barcodes for each sample. Finally, enrichment PCR was carried out to enrich those DNA fragments that have adapter molecules on both ends and to amplify the amount of DNA in the library.

#### Sequencing

Sequence-libraries of each sample were equimolarly pooled and sequenced on Illumina HiSeq 4000 (2 lanes, 75 bp, Single Reads) at the VIB Nucleomics core (www.nucleomics.be).

#### Preprocessing

Low quality ends and adapter sequences were trimmed off from the Illumina reads with FastX 0.0.14 and Cutadapt 1.15 [30] (http://hannonlab.cshl.edu/fastx_toolkit/index.html). Subsequently, small reads (length < 35 bp), polyA-reads (more than 90 % of the bases equal A), ambiguous reads (containing N), low-quality reads (more than 50 % of the bases < Q25) and artifact reads (all but three bases in the read equal one base type) were filtered using FastX 0.0.14 and ShortRead 1.40.0 [31]. With Bowtie2 2.3.3.1 we identified and removed reads that align to phix_illumina [32].

#### Mapping

The preprocessed reads were aligned with STAR aligner v2.5.2b to the reference genome of Homo sapiens (GRCh38) [33]. Default STAR aligner parameter settings were used, except for ‘--outSAMprimaryFlag OneBestScore --twopassMode Basic --alignIntronMin 50 --alignIntronMax 500000 --outSAMtype BAM SortedByCoordinate’. Using Samtools 1.5, reads with a mapping quality smaller than 20 were removed from the alignments [34].

#### Counting

The number of reads in the alignments that overlap with gene features were counted with featureCounts 1.5.3 [35]. Following parameters were chosen: -Q 0 -s 2 -t exon -g gene_id. We removed genes for which all samples had less than 1 count-per-million. Raw counts were further corrected within samples for GC-content and between samples using full quantile normalization, as implemented in the EDASeq package from Bioconductor [36]. All fastq and supplementary files were uploaded to NCBI-GEO under accession number GSE162892.

#### Differential Gene Expression

With the EdgeR 3.24.3 package of Bioconductor, a negative binomial generalized linear model (GLM) was fitted against the normalized counts [37]. We did not use the normalized counts directly, but worked with offsets. Differential expression was tested for with a GLM likelihood ratio test, also implemented in the EdgeR package. The resulting p-values were corrected for multiple testing with Benjamini-Hochberg to control the false discovery rate [38].

#### Gene Set Enrichment Analysis

To detect enriched functional gene sets, we used the online tool Webgestalt (http://www.webgestalt.org/) with gene sets from the “biological process” gene ontology functional database. Genes were ranked by the signed log of the P values from the differential expression analysis. The sign comes from the differentially expressed log2-values. We used the HGNC symbols for gene identifiers. We limited our analysis to gene sets with less than or equal to 500 genes and more than or equal to 20 genes.

#### Inclusion Data Zhang et al., Li et al., Tchieu et al.

Fastq files from Zhang et al. [39], Li et al. [21] and Tchieu et al. [22] were downloaded from the European Nucleotide Archive. Quality control of raw reads was assessed with FastQC version 0.11.5. Cutadapt version 1.9.1 was used to trim residual adapter sequences, bases with Phred-scores lower than 20, poly A {10} tail sequences, and reads shorter than 20 bases. STAR version 020201 was used to assemble a genome index, using the human genome fasta files (GRCh38.92) and GTF file from Ensembl. Subsequently, STAR version 020201 was used to align the trimmed reads to the human genome. Raw read count matrices were generated with FeatureCounts (from RsubRead). The raw count matrices from Zhang et al. and from this study were merged into one matrix. Merged exon lengths were calculated with GTFtools and subsequently used for TPM and log2 (TPM + 1) calculation. The Combat algorithm from the sva R package was used for batch effect correction. Principal Component Analysis was performed on the corrected log2 (TPM + 1) values using the R stats package.

### Glutamate Uptake

DIV33-48 iSOX9-astrocytes and fHA were seeded in matrigel-coated plates at 20 000 cells/cm^2^. The next day, medium was replaced with DMEMF12 Glutamax™ medium which contains 50 µM glutamate, supplemented with 1X N2, 1X sodium pyruvate, 0.5 mM N-acetyl-cysteine, 0.1 mM dbcAMP, 10 ng/ml CNTF, 10 ng/ml BMP4 and 5 ng/ml HB-EGF. After 24, 48 and 72 h, medium was collected for mass spectrometry.

Metabolite extraction was performed in a mixture ice-dry ice as described before [40, 41]. A standard curve from 0 to 100 µmol/L of glutamic acid was prepared to determine the uptake/secretion of glutamic acid in media samples. A volume of 20 µL of media samples and standards were resuspended in 800 µL of methanol/water (5/3) (v/v) containing 0.6 µg/ml of glutaric acid as internal standard. Subsequently, a volume of 500 µL of precooled chloroform was added to each sample. Samples were then vortexed for 10 min at 4 °C and centrifuged for other 10 min (max. speed, 4 °C). After centrifugation, metabolites were separated in two phases: polar metabolites in the methanol/water (upper) phase and the lipid fraction in the chloroform (lower) phase. Following metabolites separation, polar metabolite phase containing glutamic acid was dried at 4 °C overnight using a vacuum concentrator. The samples were derivatized and measured as described before [42]. Briefly, polar metabolites were derivatized in 20 µL of 20 mg/ml methoxyamine in pyridine per sample for 90 min at 37 °C. Subsequently, 15 µL of N-(tert-butyldimethylsilyl)-N-methyl-trifluoroacetamide, with 1 % tert-butyldimethylchlorosilane were added to 7.5 µL of each derivative and incubated for 60 min at 60 °C.

The metabolites were analyzed by gas chromatography (7890 A GC system) coupled to mass spectrometry (5975 C Inert MS system) from Agilent Technologies. Metabolites were separated with a DB35MS column (30 m, 0.25mm, 0.25 μm) using a carrier gas flow of helium fixed at 1 ml/min for the analysis of polar metabolites. A volume of 1 µL of sample were injected in splitless mode with an inlet temperature set at 270 °C. For the detection of polar metabolites, the gradient started at 100 °C for 1 min ramped to 105 °C at 2.5 °C/min, then to 240 °C at 3.5 °C/min and finally to 320 °C at 22 °C/min. For the measurement of metabolites by mass spectrometry, the temperatures of the quadrupole and the source were set at 150 and 230 °C, respectively. An electron impact ionization fixed at 70 eV was applied and a selected-ion monitoring (SIM) mode was used for the measurement of polar metabolites.

After the acquisition by GC-MS, a inhouse Matlab M-file was used to extract mass distribution vectors and integrated raw ion chromatograms. The natural isotopes distribution were also corrected using the method developed by Fernandez et al., 1996 [43]. The peak area was subsequently normalized to the internal standard: glutaric acid.

### ELISA

DIV33-48 iSOX9-astrocytes from three independent differentiations and fHA were exposed for 5 days to either 10 ng/ml TNF-α (Peprotech), 100 ng/ml IL-6 (Peprotech), 10 ng/ml IL-1β (Peprotech) or the combination of TNF-α and IL-1β. In addition, an untreated condition was taken into account. After 5 days, medium was collected in duplicates for the iSOX9-astrocytes and in triplicate for the fHA. The following analytes were measured using the LEGENDplex™ bead-based immunoassay according manufacturer’s instructions: GM-CSF, HGF, IFN-α, IFN-β, IL-10, IL-12, IL-1α, IL-1β, IL-23, IL-6, LIF, PDGF-AA, TNF-α.

### Calcium Imaging

DIV33-48 iSOX9-astrocytes and fHA were plated in a round 4-chamber cover slip (100 000 cells/chamber) one week prior to the measurements. On the day of the recordings, cells were loaded with 1.25 µM Fura-2 (Eurogentec) in modified Krebs solution (150 mM NaCl, 5.9 mM KCl, 1.2 mM MgCl_2_, 11.6 mM HEPES (pH 7.3), 11.5 mM glucose and 1.5 mM CaCl_2_; all purchased from Sigma) at RT in the dark. After 30 minutes, the cells were washed twice with the above modified Krebs solution followed by a 30’ incubation/de-esterification step at RT in the modified Krebs solution. During the recordings, an automated suction system was positioned to ensure complete medium change upon manual addition of the different stimuli. Each measurement was started by addition of 3 mM BAPTA (VWR) in the modified Krebs solution lacking 1.5 mM CaCl_2_, to chelate extracellular Ca^2+^. One minute later, the different stimuli, solubilized in modified Krebs solution without CaCl_2_ supplemented with 3 mM BAPTA, were added and responses were measured. For quantifying ATP (20 µM, Roche) and Ach (20 µM, Sigma) responses, the number of responding cells were counted as well as the AUC was determined. For GPN (200 µM, Abcam), FCCP (10 µM, Abcam), thapsigargin (2 µM, Alomone labs) and ionomycin (2 µM, Alomone labs), the area under the curve for each trace was calculated. Changes in Fura-2 fluorescence intensity were measured at 510 nm while alternating excitation at 340 nm and 380 nm utilizing a Zeiss Axio Observer Z1 Inverted Microscope equipped with a 20× air objective and a high-speed digital camera (Axiocam Hsm, Zeiss, Jena, Germany). Traces are shown as the ratio of emitted fluorescence of Fura-2 (F340/F380).

### Patch Clamping of Astrocytes

DIV48 iSOX9-astrocytes and fHA were plated on a matrigel-coated 3 cm coverslip at a density of 8000 cells/cm^2^ and whole-cell voltage clamp recordings were performed. The intracellular pipette solution contained 136 mM KCl, 18 mM HEPES, 4 mM Na-ATP, 4.6 mM MgCl_2_, 4 mM K_2_-ATP, 15 mM creatine phosphate, 1 mM EGTA and 50 U/mL phospocreatine kinase (300 mOsm, pH 7.30). The extracellular solution used during recordings contained 140 mM NaCl, 2.4 mM KCl, 2 mM CaCl_2_, 2 mM MgCl_2_, 10 mM HEPES, 10 mM glucose (300 mOsm, pH 7.30). Cultured astrocytes were whole-cell voltage clamped at -70 mV using a double EPC-10 amplifier (HEKA Elektronik, Lambrecht/Pfalz, Germany) under control of Patchmaster v2 × 32 software (HEKA Elektronik). Currents were recorded at 20 Hz and low-pass filtered at 3 kHz when stored. Pipettes were pulled using a Sutter P-1000 and resistance ranged from 3 to 5 MΩ. The series resistance was compensated to 75–85 %. Cells with series resistances above 15 MΩ were excluded for analysis. I/V-traces were generated using a step protocol (-120 to + 120 mV, 20 mV steps) from a holding potential of -70 mV. Na^+^ and K^+^ currents were measured at the beginning and end of the voltage steps, respectively. Resting membrane potential (Vm) was measured after the establishment of whole-cell configuration (in current clamp, Iholding = 0 pA). All recordings were performed at room temperature.

### Multi-electrode Array (MEA) Experiments

Human iNGN2-neurons, generated by 4 days of doxycycline (2 µg/ml) addition to iNGN2-PSCs in NMM, were co-cultured with DIV40 iSOX9-astrocytes from 4 independent differentiations in 1:1 ratio and plated in NMM without doxycycline onto 48‐well MEA plates (Axion BioSystems) pre-coated with 0.1 % polyethyleneimine (Sigma). Spontaneous network activity was recorded for 5 minutes 37 days after seeding using the Axion Biosystems Maestro MEA at 37 °C and 5 % CO_2_. Data analysis was performed using AxIs software (Axion Biosystems Inc.). Active electrodes, (AEs; 16 electrodes per well) were defined as electrodes averaging more than 5 spikes per minute. Active wells were defined as those that have more than 30 % of active electrodes. All wells that did not comply with these criteria were discarded. The threshold for spike detection was defined as ≥ 5.5-fold the standard deviation of the RMS (root mean square) noise. To be able to calculate the synchronicity of the neuronal culture, at least 25 % of the total electrode in a well should participate to the network bursts.

### Statistical Analysis

All cell culture experiments were performed in triplicate (three independent differentiations; except for the lentiviral screen where only two independent differentiations were carried out) and values are represented as mean ± SEM. For comparing means comprising one categorical variable, One-way ANOVA was performed with Bonferroni correction for multiple comparisons. Similarly, Two-way ANOVA was used if two categorical variables were taken into account and was performed with Bonferroni correction. Statistical significance was reached when p ≤ 0.05. All data was processed via GraphPad Prism version 9.0.0 (GraphPad Software, San Diego, California, USA).

## Results

### Six Days of *SOX9* Overexpression in iPSC-derived NPCs Upregulates the Expression of Astrocyte-specific Proteins

To test the hypothesis that overexpression of *SOX9* alone, without the co-factors *NFI-A* and *NFI-B*, at the NPC stage would be sufficient to induce an astrocyte fate, we performed a preliminary lentiviral vector-based screen. NPCs were generated from PSCs by dual SMAD inhibition. On DIV12, iPSC-derived NPCs were transduced with lentiviral vectors encoding for doxycycline-inducible *SOX9*, *NFI-A* or *NFI-B*, either alone or in combination. Inducible overexpression of the TFs was confirmed via RT-qPCR (Figure S1). Flow cytometry (Figure S2b) and RT-qPCR (Figure S2c) one week after transduction demonstrated that overexpression of *SOX9* alone was sufficient to induce an astrocyte phenotype at the transcript and protein level. Early neuronal transcripts (*TUBB3, PAX6, NESTIN*) were not induced and pluripotency transcript *OCT4* did not increase after lentiviral transduction of any of the combinations (Figure S2c).

To ensure that all NPCs contain a single copy of the transgene and to avoid the theoretical possibility of insertional mutagenesis caused by lentiviral transduction, we next recombined a cassette encoding for *SOX9* controlled by a TET-ON promotor via recombinase-mediated cassette exchange (RMCE) in the *AAVS1* locus as described in Ordovas et al., [29]. First, we introduced by homologous recombination using zinc finger nucleases (ZFNs) a GFP-expressing cassette flanked by two FRT-sites into the *AAVS1* locus of iPSCs. We confirmed specific integration via flow cytometry, PCR and Southern Blot (Figure S2d and Figures S3a-b). Afterwards, we recombined with Flippase (Flp) an FRT-flanked cassette containing *SOX9* controlled by a TET-ON promoter. After RMCE, GFP expression disappeared (Figure S2d) and doxycycline addition to the cells induced *SOX9* transcript levels (Figure S2e). Genome-edited stem cells were tested by array comparative genomic hybridization (aCGH) and showed no genome wide mutations (Figure S3c). In addition, embryoid body (EB) formation and Scorecard® analysis demonstrated pluripotency of the genome-engineered iSOX9-iPSCs (SIGi001-A-20) (Figure S3d).

**Fig. 1:**
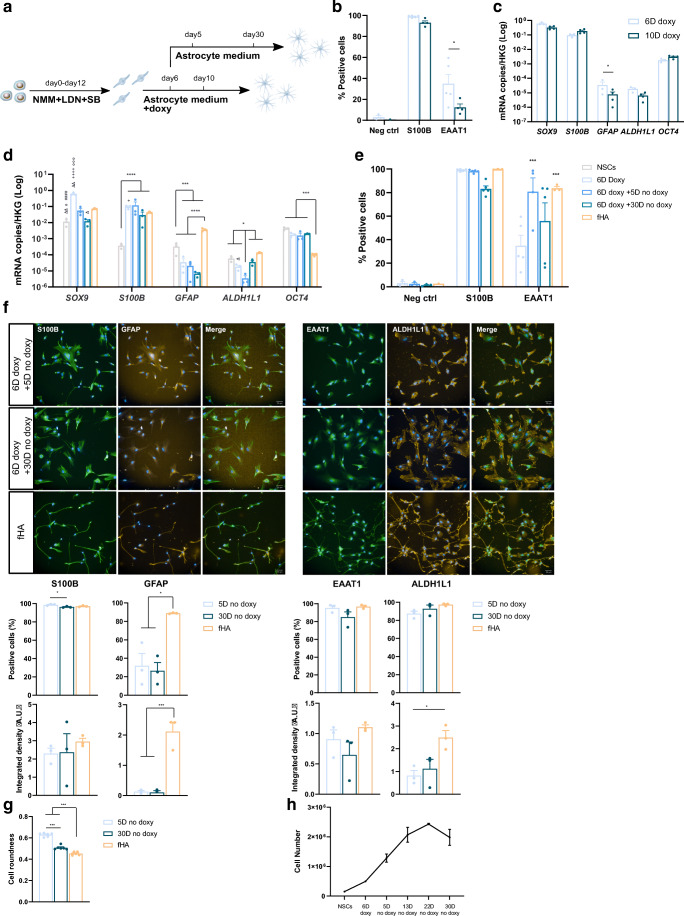
Six days of *SOX9* overexpression in iSOX9-derived NPCs upregulates the expression of astrocyte-specific proteins. **a** Overview optimization of iSOX9-astrocyte differentiation. First, the number of days to overexpress *SOX9* were tested by comparing 6 and 10 days of doxycycline exposure. Next, the effect of removing doxycycline was assessed. **b** Flow cytometry to assess the percentage of S100B and EAAT1 expressing cells after exposing iSOX9-derived NPCs for 6 and 10 days to doxycycline (N = 4–5 independent differentiations; *p < 0.05). **c** RT-qPCR of *SOX9*, *S100B*, *GFAP*, *ALDH1L1* and *OCT4* transcripts after exposing iSOX9-derived NPCs for 6 and 10 days to doxycycline (N = 3–4 independent differentiations; *p < 0.05).  **d** RT-qPCR of *SOX9*, *S100B*, *GFAP*, *ALDH1L1* and *OCT4* transcripts at different time points throughout the optimized differentiation protocol compared to fHA. (N = 3 independent differentiations; ^Δ^p < 0.05 versus fHA, ^####^p < 0.0001 versus 6D doxy, °p < 0.05 versus 5D no doxy, ^+^p < 0.05 versus 30D no doxy). **e** Flow cytometry to assess the percentage of S100B and EAAT1 expressing cells at different time points throughout the optimized differentiation protocol compared with fHA (N = 3–5 independent differentiations;***p < 0.001 versus 6D doxy). **f** Representative immunofluorescence images of fHA and iSOX9-astrocytes at 5 and 30 days after stopping doxycycline treatment for S100B (green), GFAP (red), EAAT1 (green) and ALDH1L1 (red) (scale bar: 50 μm). Quantification of the percentage of positive cells and integrated density was performed using Columbus Image analysis software (PerkinElmer) (N = 3 independent differentiations; *p < 0.05, ***p < 0.001). **g** Cell roundness quantification of fHA and iSOX9-astrocytes after stopping doxycycline treatment for 5 and 30 days (N = 3 independent differentiations; ***p < 0.001). **h** Quantification of cell numbers throughout iSOX9-astrocyte differentiation (N = 3 independent differentiations). All data represented as mean ± SEM

Using flow cytometric analysis for S100B and EAAT1 and transcript analysis of astrocyte-related genes, we tested how many days of doxycycline-mediated *SOX9* overexpression was necessary and sufficient to create iSOX9-astrocytes (Fig. 1a-c). These studies demonstrated that a stable astrocyte fate was already obtained after 6 days of forced *SOX9* overexpression. Specifically, when doxycycline was removed on day 6 and NPC-progeny reassessed by RT-qPCR 5 and 30 days later, *S100B* transcript levels remained steady and day 30 levels were similar to those found in cultured fetal human astrocytes (fHA) (Fig. 1d). As expected, removal of doxycycline reduced transcript levels of *SOX9* (Fig. 1d). Transcript levels for *GFAP* were lower in iSOX9-astrocytes compared to fHA on day 6 of doxycyline treatment as well as 5 and 30 days after removal of doxycycline (p < 0.0001) (Fig. 1d). Flow cytometry and immunostaining demonstrated that the fraction of cells positive for S100B and EAAT1 was similar to fHA in iSOX9-astrocytes 5 and 30 days after stopping doxycycline (Fig. 1e, f). Immunostaining also demonstrated that as for fHA, the majority of iSOX9-astrocytes stained positive for ALDH1L1 (Fig. 1f). Consistent with the RT-qPCR results, significantly fewer day 5 and day 30 iSOX9-astrocytes stained positive for GFAP and GFAP intensity was lower compared to fHA (p < 0.05) (Fig. 1f). Morphological analysis demonstrated that the roundness of iSOX9-cells decreased between 5 and 30 days of doxycycline removal, but fHA roundness was even smaller compared with day 30 iSOX9-astrocytes (p < 0.001) (Fig. 1g). A progressive decrease in proliferation of iSOX9-astrocyte progeny was observed from ± day 20 after stopping doxycycline onwards (Fig. 1h). Per iPSC, 105.05 ± 11.43 iSOX9-astrocytes could be generated. Moreover, iSOX9-astrocytes could be cryopreserved and did not lose their astrocytic phenotype upon thawing (Figure S4). In conclusion, a nearly 100 % homogenous population of S100B and EAAT1 positive astrocyte-like cells can be generated by forced overexpression of *SOX9*, as the sole TF, for only 6 days in iPSC-derived NPCs.

### iSOX9-astrocytes Display Functional Glutamate Handling, Electrophysiological Properties, Cytokine/growth Factor Secretion as well as Intracellular Calcium Responses

**Fig. 2:**
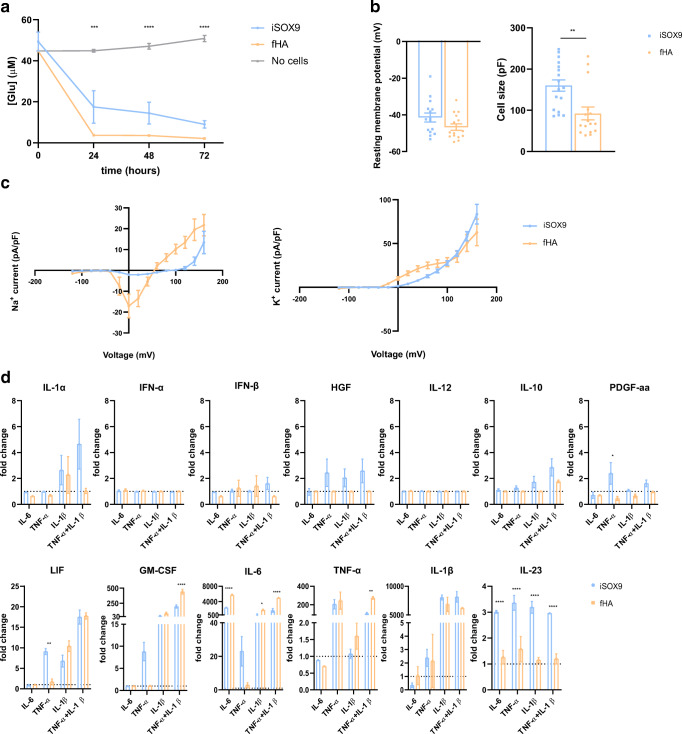
Functional characterization of iSOX9-astrocytes based on glutamate transport, patch clamp measurements and cytokine secretion.** a** Glutamate concentration was measured via mass spectrometry from the medium conditioned for 24, 48 and 72 h by iSOX9-astrocytes (N = 8 independent differentiations with two technical replicates for each differentiation) or fHA (N = 4). In addition, medium with no cells was taken into account as negative control (N = 4) (***p < 0.001 versus fHA and iSOX9). **b** Resting membrane potential and cell capacitance (reflecting cell size) measurements of fHA (N = 15 cells of 3 independent differentiations) and iSOX9-astrocytes (N = 15 cells of 3 independent differentiations) (**p < 0.01). **c** Na^+^ and K^+^ current measurements via patch clamp at voltages between − 120 mV and + 160 mV of fHA (N = 14) and iSOX9-astrocytes (N = 17 cells of 3 independent differentiations). **d** After exposing iSOX9-astrocytes (N = 3 independent differentiations with two technical replicates for each differentiation) and fHA (N = 3) for 5 days to IL-6, TNF-α, IL-1β or the combination of TNF-α and IL-1β, the concentration of IL-1α, IFN-α, IFN-β, HGF, IL-12, IL-10, LIF, GM-CSF, IL-6, TNF-α, IL-1β, PDGFaa and IL-23 was measured in the medium using a bead-based immunoassay. Each time, the measured concentration was divided by the concentration of untreated cells (*p < 0.05;**p < 0.01;****p < 0.0001). All data represented as mean ± SEM

The function of iSOX9-astrocytes was assessed between 15 and 30 days after doxycycline removal (DIV33-48) in comparison with fHA as positive control. As astrocytes are responsible for glutamate removal from the synapse to prevent glutamate-induced excitotoxicity, glutamate handling was assessed [44]. We incubated iSOX9-astrocytes and fHA with medium containing 50 µM glutamate and measured glutamate concentration in the medium after 24, 48 and 72 h by mass spectrometry. No significant differences in glutamate uptake were observed between iSOX9-astrocytes and fHA (Fig. 2a).

**Fig. 3:**
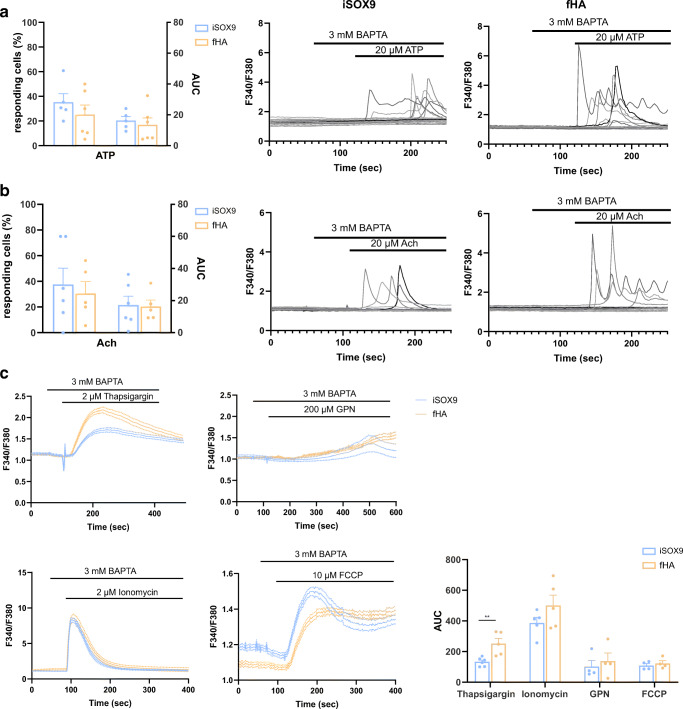
iSOX9-astrocytes show comparable calcium responses as fHA. Calcium responses (plotted as F340/F380 ratio) measured in Fura-2-loaded iSOX9-astrocytes and fHA in the presence of extracellular Ca^2+^-chelating agent BAPTA (3 mM), ensuring only Ca^2+^ release from internal Ca^2+^ stores is measured. **a** After stimulating fHA and iSOX9-astrocytes with ATP, they show a similar calcium response measured by Fura2 fluorescence emission (N = 6 independent differentiations). **b** After stimulating fHA and iSOX9-astrocytes with Ach, they show a similar calcium response measured by Fura2 fluorescence emission (N = 6 independent differentiations). **c** iSOX9-astrocytes and fHA show a similar calcium response after stimulation with GPN, FCCP and ionomycin measured by Fura2 fluorescence emission. A significantly higher calcium response is observed in fHA compared to iSOX9-astrocytes after thapsigargin stimulation (**p < 0.01) (N = 6 independent differentiations). All data represented as mean ± SEM

Patch clamp experiments were performed to characterize the electrophysiological properties of iSOX9-astrocytes in comparison with fHA. No differences were observed in resting membrane potential between iSOX9-astrocytes and fHA (Fig. 2b). However, cell capacitance measurements revealed a significant bigger cell size of iSOX9-astrocytes compared to fHA (p < 0.01) (Fig. 2b), in line with the morphometric analysis shown in Fig. 1g. As functional astrocytes express voltage-gated channels, we also measured Na^+^ and K^+^ currents at voltages ranging from − 120mV until + 160mV. Results were corrected for cell size. Although we observed Na^+^ currents in iSOX9-astroyctes, these were less pronounced compared to fHA (Fig. 2c). Outward K^+^ currents were comparable between the iSOX9-astrocytes and fHA (Fig. 3c). Both Na^+^ and K^+^ currents were significantly lower in both iSOX9-astrocytes and fHA compared to what is commonly measured in neurons [45], further demonstrating that iSOX9-astrocytes do not contain neural contaminants.

We also assessed the response of iSOX9-astroyctes to inflammatory stimuli (IL-6, TNF-α, IL-1β or the combination of TNF-α and IL-1β). After 5 days of stimulation, secretion of different cytokines and growth factors was assessed by a bead-based multiplex immunoassay for pro-inflammatory (GM-CSF, TNF-α, IL-1α, IL-1β, IL-6, IL-12 and IL-23) and anti-inflammatory (IFN-α, IFN-β, IL-10, HGF, LIF and PDGFaa) cytokines/growth factors. Data is presented as the fold change compared to untreated iSOX9-astrocytes or fHA. In general the cytokine/growth factor levels secreted by iSOX9-astrocytes and fHA were similar. Specifically, no IL-1α, IFN-α, IFN-β, HGF, IL-12 and IL-10 response was observed in either iSOX9-astrocytes or fHA in response to any of the inflammatory stimuli. Exposure to IL-1β, with or without TNF-α, induced secretion of LIF, IL-6 and GM-CSF by both iSOX9-astrocytes and fHA (Fig. 2d). Increased secretion of IL-23 was observed to all stimuli in iSOX9-astrocytes only (p < 0.0001) and TNF-α treatment induced secretion of the protective growth factors LIF and PDGFaa by iSOX9-astrocytes, but not fHA (p < 0.05) (Fig. 2d). Finally, GM-CSF and IL-6 secretion induced by IL-1β + TNF-α was higher in fHA compared to iSOX9-astrocytes (p < 0.0001)(Fig. 2d).

As astrocyte communication is governed by Ca^2+^ signaling, we also compared single cell Ca^2+^ responses of iSOX9-astrocytes and fHA. First, two physiological inducers ATP and acetylcholine (Ach) were used to trigger intracellular Ca^2+^ release. The fraction of iSOX9-astrocytes responding to ATP and Ach and the corresponding area under the curve (AUC) were similar to that of fHA (Fig. 3a, b). Also histamine and caffeine were used, but as they did not induce Ca^2+^ responses in either iSOX9-astrocytes or fHA, data is not shown. Next, thapsigargin and ionomycin were used as triggers in order to measure the endoplasmic reticulum (ER) Ca^2+^ store content and total cellular Ca^2+^ content respectively. Thapsigargin induces the depletion of the ER Ca^2+^ store by blocking Ca^2+^ reuptake via the sarco/endoplasmic reticulum Ca^2+^ ATPase. Thapsigargin treatment induced a significantly lower Ca^2+^ response in iSOX9-astrocytes compared to fHA (p < 0.01). However, the total cellular Ca^2+^ release triggered by ionomycin was similar in both astrocyte types (Fig. 3c). To assess Ca2^+^ release related to other organelles besides the ER, cells were also exposed to glycyl-L-phenylalanine 2-naphthylamide (GPN) and carbonyl cyanide 4-(trifluoromethoxy)phenylhydrazone (FCCP). GPN triggers intracellular Ca^2+^ release by inducing the rupture of lysosomal membranes or by increasing both lysosomal and cytosolic pH resulting in Ca^2+^ release in an ER-dependent manner [46]. FCCP exposure leads to collapse of the mitochondrial membrane potential and subsequent mitochondrial Ca^2+^ release which can then be amplified by ER Ca^2+^ release or by Ca^2+^ influx over the plasma membrane [47]. Exposure of iSOX9-astrocytes to both stimuli induced similar Ca^2+^ responses as seen for fHA (Fig. 3c).

Thus, iSOX9-astroyctes have the ability to handle glutamate, have electrophysiological properties, respond to inflammatory stimuli and show intracellular Ca^2+^ responses that are in general similar to fHA.

### Transcriptional Profiling of iSOX9-astrocytes Reveals Maturation After Stopping Doxycycline Treatment

**Fig. 4:**
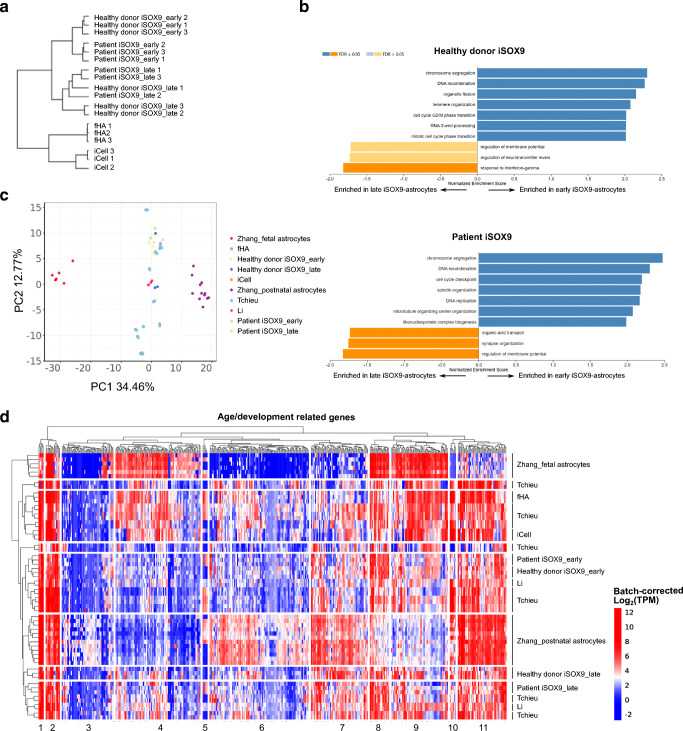
RNASeq analysis of iSOX9-astrocytes reveals maturation after stopping doxycycline treatment and resemblance to isolated postnatal astrocytes.** a** Clustering of the iSOX9-astrocytes 5 (early) and 30 days (late) after stopping doxycycline treatment using a healthy donor-derived iPSC line and iPSCs derived from an ALS patient (each time N = 3 independent differentiations). Commercially available primary human fetal astrocytes (fHA) (N = 3) and iPSC-derived astrocytes (“iCell”) (N = 3) were also included. **b** Ranked GSEA of the differentially expressed genes between early and late iSOX9-astrocytes. Biological processes with a positive normalized enrichment score (NES) (blue bars) are enriched in the early iSOX9-astrocytes, while the processes with a negative NES (orange bar) are enriched in the late iSOX9-astrocytes. This analysis was performed for both the healthy donor and patient-derived iPSC-derived cells. **c** PCA plot using only the genes that showed at least a 16-fold increased or decreased expression between the postnatal and fetal samples of Zhang et al., of the previous samples mentioned in panel (**a**) combined with RNASeq data from isolated postnatal and fetal human astrocytes from Zhang et al. and the iPSC-derived astrocytes from Li et al. and Tchieu et al. **d** Expression values of the genes that showed at least a 16-fold increased or decreased expression between the postnatal and fetal samples of Zhang et al. Those genes can be divided into 11 clusters and are considered important for development/age

We performed RNA sequencing on iSOX9-astrocytes 5 (early; DIV23) and 30 (late; DIV48) days after stopping doxycycline treatment. Aside from the healthy donor derived SIGi001-A-20 iSOX9-astrocytes, we also included early and late iSOX9-astrocytes from an ALS patient-derived iPSC line (as described in Guo et al., [48]), genome-engineered in the same way to allow forced expression of *SOX9*. We also included commercially available iPSC-derived astrocytes (“iCell” astrocytes) and fHA. Hierarchical clustering demonstrated that all iSOX9-astrocytes (independent of harvest day and donor origin) clustered separately from iCell astrocytes and cultured fHA. Within the group of iSOX9-astrocytes, early and late astrocytes clustered separately, again independent of the donor origin of the iPSC (Fig. 4a). Differential gene expression analysis was used to identify genes that were signficanly less or more expressed in early versus late iSOX9-astrocytes. Gene set enrichment analysis (GSEA) identified proliferation-related processes to be enriched in early astrocytes independent of the iPSC origin (Fig. 4b). Late iSOX9-astrocytes generated from both iPSC lines displayed an enrichment of astrocyte-related biological functions such as synapse organization and regulation of membrane potential and neurotransmitter levels. The GSEA analysis is therefore indicative of maturation of iSOX9-astrocytes after doxycycline removal.

To assess how well iSOX9-astrocytes approximate primary uncultured human astrocytes, we used transcriptome data of human fetal and postnatal brains published by Zhang et al. [39]. We also compared iSOX9 transcriptome data with data from Li et al. who created astrocytes by overexpression of SOX9 and NFIA directly in PSCs [21], and Tchieu et al. who overexpressed only NFIA directly in PSC-derived long-term neural stem cells [22]. Principle component analysis (PCA) of all genes is shown in Figure S5. As this PCA only captured 24.55 % of genes in principle components 1 and 2, we next identified genes that were at least 16-fold differentially expressed between human primary brain-derived fetal or postnatal astrocytes from Zhang et al. This selection should identify astrocyte-specific genes that are linked to age and/or the development state of astrocytes, which is the factor we are interested in for comparing the different astrocyte transcriptomes. When the PCA was repeated based on these 443 genes (Fig. 4c), we again found that culture-expanded fHA as well as iCell astrocytes clustered separately from iSOX9-astrocytes from both donors. This analysis also demonstrated that all astrocytes generated *in vitro* by TF overexpression clustered together, and this in between freshly isolated fetal and postnatal astrocytes (Fig. 4c). Hierarchical clustering analysis of all samples (Fig. 4d) substantiated the notion that iSOX9-astrocytes continue maturing when maintained longer in culture. Late iSOX9-astrocytes (both from healthy donor and patient) clustered closer together with postnatal than prenatal freshly isolated astrocytes. This was exemplified by lower levels of *MKI67, TOP2A, TPX2 and NUSAP1*, genes known to be highly expressed in astrocyte progenitor cells but downregulated upon maturation, both in late iSOX9-astrocytes and postnatal astrocytes (Figure S6) [1]. Conversely, the mature astrocyte markers *EAAT1*, *ALDH1L1* and *AQP4* were more highly expressed in late compared to early iSOX9-astrocytes and equally expressed compared to postnatal astrocytes (Figure S6). Hierarchical clustering analysis of the 443 genes identified 11 clusters (Fig. 4d). Clusters 4 and 9 represent genes involved in development and proliferation-related processes (e.g. “telencephalon development” and “nuclear division”) and were expressed at higher levels in primary fetal astrocytes but at lower levels in postnatal isolated as well as iSOX9-astrocytes. Conversely, genes of cluster 6 and 11 comprising functional processes (e.g. “calcium ion transport” and “amino acid transport”) were more highly expressed in postnatal compared with fetal isolated astrocytes, and remained lower expressed in all TF-guided PSC-astrocyte populations (Fig. 4d and Figure S7a, S7b ).

### iSOX9-astrocytes Support iPSC-derived Neuron Maturation

**Fig. 5:**
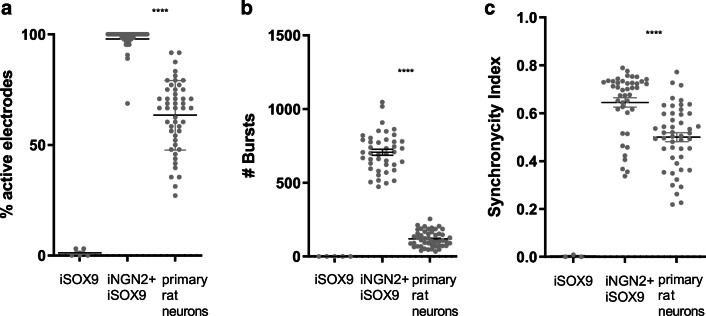
iSOX9-astrocytes support iPSC-derived neuron maturation. DIV4 iNGN2-PSC-derived neurons were co-cultured 1:1 with DIV40 iSOX9-astrocytes for 37 days and electrical activity was measured using MEA plates (16 electrodes per well of a 48-well plate). As control, iSOX9-astrocytes alone and rat primary cortical neurons were also evaluated. Depicted are the % of active electrodes (**a**), number of bursts (**b**) and synchronicity index (**c**). Data is represented as mean ± SEM of N = 3–4 independent experiments

The most conclusive approach to assess the functional quality of human iPSC-derived astrocytes is by performing electrophysiological studies of iPSC-derived neurons co-cultured with iPSC-derived astrocytes in a fully humanized assay set-up. To test the ability of iSOX9-astrocytes to support neuronal maturation and functioning, we co-cultured them with iNGN2-neurons (as described in a recently submitted manuscript by Shih et al.). iNGN2-derived NPCs were co-cultured together with DIV40 iSOX9-astrocytes on multi-electrode arrays (MEA) and readouts are shown after 37 days of co-culture (= DIV77). iSOX9-astrocyte mono-cultures were used as negative control, which did not display detectable electrical activity. We also added the comparison with primary rat neurons as the gold-standard reference used in electrophysiological studies. It has been previously shown that iNGN2-neurons on their own do not display electrical activity [49]. However, when co-cultured with iSOX9-astrocytes, we observed that the percentage of active electrodes and number of bursts significantly exceeded that of primary rat neurons (p < 0.0001) (Fig. 5a, b). Importantly, the synchronicity index reflecting neural network formation was also significantly higher in iNGN2-neurons co-cultured with iSOX9-astrocytes than in primary rat neurons (p < 0.0001) (Fig. 5c).

## Discussion

In this study, we present a fast and efficient protocol to create human astrocytes from PSCs that support robust synchronous activity of PSC-derived iNGN2-neurons. This all-human PSC-derived astrocyte-neuron co-culture system represents an asset for high-throughput neural drug discovery studies, by which animal use may be reduced. However, complete removal of laboratory animals from research is not possible yet as animal models can capture the complex interactions in the neuronal system, which is not the case for these co-cultures.

The protocol to generate large numbers of functional human astrocytes depends on the overexpression of *SOX9*, as the sole TF, in DIV12 PSC-derived NPCs. Six days after doxycycline-induced *SOX9* overexpression, a nearly 100 % pure EAAT1, ALDH1L1 and S100B positive cell population was obtained that could be further stably expanded for at least 30 days. iSOX9-astrocytes could be cryopreserved without loss of function after thawing as all mentioned functional read-outs were performed on both fresh and cryopreserved cells. Functionally, iSOX9-astrocytes displayed similar functional properties as cultured primary fetal human astrocytes (fHA). Transcriptionally, iSOX9-astrocytes showed similarities with postnatal brain astrocytes. As indicated above, iSOX9-astrocytes robustly supported the electrophysiological maturation of iNGN2-neurons.

Other groups already exploited TF-guided astrocyte differentiation to generate astrocytes from human PSCs [20–22]. Canals et al. transduced undifferentiated PSCs with lentiviral vectors (DIV1) encoding for TET-inducible *SOX9* and *NFI-B*, generating iAstrocytes after 21 days of doxycycline-induced overexpression of both TFs (DIV2-22). Although this protocol is shorter compared to ours (DIV33-48), the use of lentiviral vectors has the drawback that the copy number of either of the two viral vectors per cell likely varies. Moreover, silencing of viral vector integrants in longer term culture PSCs has been seen. Hence, because of variable vector integration and/or silencing, additional purification steps might be necessary at the end of differentiation. In addition, random integration of the vectors into the genome may lead to variable gene expression and viral vectors can cause insertional mutagenesis. Moreover, the protocol described by Canals et al. yielded only 1.8 iAstrocytes per starting PSC, while the method described here yields ± 105 iSOX9-astrocytes per PSC. In a second study, Li et al. created PSCs wherein TET-inducible cDNAs for *NFI-A* and *SOX9* were stably integrated into the *AAVS1* locus of PSCs. In this study too, activation of the TFs via doxycycline was performed at the PSC stage creating astrocyte progeny 52 days later, which is longer than the iSOX9-astrocyte protocol described here. In a third paper, Tchieu et al. also used a single TF overexpression system, by integrating a TET-inducible *NFI-A* in the *AAVS1* locus of PSCs. After 20 days of neural induction (DIV20), NFI-A was induced by doxycycline addition for 10 days (DIV30), with a stable cell phenotype for another 30–60 days (DIV60-120) after doxycycline removal. In line with Tchieu et al., we observed that fewer than 50 % of iSOX9-astrocytes expressed GFAP, which is significantly lower than what is seen for cultured fHA. However, *GFAP* gene expression in fHA is also considerably higher compared to the expression in freshly isolated fetal or adult astrocytes (Figure S6). As GFAP is expressed at low(er) levels in quiescent adult brain astrocytes but induced when astrocytes are activated [50, 51], this may suggest that iSOX9-astrocytes have a less activated phenotype than fHAs. Of note, evaluation of the RNASeq studies perfomed by Li et al. and Tchieu et al. demonstrated similar *NFI-A* and *SOX9* expression levels as observed in iSOX9-astrocytes, even if we did not overexpress NFI-A as is done in either published studies (data not shown). This is in line with murine development studies demonstrating that SOX9 induces *NFI-A* expression and that the presence of both NFI-A and SOX9 is required for astrocyte fating and maturation [24, 25].

To characterize the iSOX9-astrocytes, we compared their functional properties with those of fHA. Similar glutamate uptake was observed and also secretion of cytokines/growth factors in response to inflammatory signals was similar for iSOX9-astrocytes and fHA which is in line with previously published studies [15, 52, 53]. Electrophysiologically, iSOX9-astrocytes displayed a similar resting membrane potential and Na^+^ and K^+^ currents as fHA, although the inward Na^+^ current was less pronounced in iSOX9-astrocytes than fHA. These patch clamp studies are consistent with the fact that the iSOX9-astrocytes are pure, and not contaminated by neurons, as the amplitude of Na^+^ and K^+^ currents measured in iPSC-derived neurons is at least 5 fold higher [45] than in the iSOX9-astrocytes. Spontaneous Ca^2+^ activity was not observed in the iSOX9-astrocytes, however intracellular Ca^2+^ release *in vitro* can be triggered in astrocytes by a number of inducers [54]. iSOX9-astrocytes as well as fHA responded in a similar manner to ATP and Ach as described before for primary rodent astrocytes [55, 56]. Compared to the differentiation protocol of Tchieu et al. in which 10 % DIV60 and 35 % DIV120 iAstrocytes responded to ATP exposure, we already observed 30 % responding iSOX9-astrocytes by DIV48. Several reports described the response of mouse or rat astrocytes to thapsigargin [57, 58], FCCP [58, 59], and ionomycin [58, 60]. We found that treatment of iSOX9-astrocytes with ionomycin, which measures the total amount of intracellular Ca^2+^ stored, was similar in iSOX9-astrocytes and fHA. However, the thapsigargin-induced response, which releases Ca^2+^ from the ER, was lower in iSOX9-astrocytes compared with cultured fHA. As GPN and FCCP treatment showed similar Ca^2+^ responses in iSOX9-astroytes and fHA, the differences observed in thapsigargin-mediated release might suggest that the amount of Ca^2+^ stored in the Golgi may be higher in iSOX9-astrocytes. However, we anticipate that the significance of this difference for the use of these iSOX9-astrocytes as model for drug development is low. ER Ca^2+^ store content is largely dependent on the amount of Ca^2+^ binding proteins present in the ER and/or overall ER size, which are expected to vary between individuals and as such may be more difficult to compare. More important is the observation that although the ER Ca^2+^ store content was lower, the iSOX9-astrocytes display similar Ca^2+^ responses to physiological agonists that trigger Ca^2+^ release from the ER, such as ATP and acetylcholine.

Whole transcriptome analysis both of normal donor and ALS iSOX9-astrocytes demonstrated over time a decreased expression of astrocyte precursor cell genes [1] and increased expression of genes involved in neurotransmitter-related processes. Therefore, we suggest that the use of iSOX9-astrocytes at later timepoints (e.g. >DIV40) may be preferred in neuronal co-culture as the proliferation rate is low and such more mature astrocytes may better functionally support neurons. Transcriptionally, iSOX9-astrocytes displayed more similarities with freshly isolated postnatal astrocytes than prenatal astrocytes as described by Zhang et al. [39], which was more pronounced for DIV48 than DIV23 iSOX9-astrocytes. However, more in-depth studies are required to confirm this.

One of the most important functions of astrocytes is to provide metabolic and trophic support towards neurons [11], which *in vitro* translates to induction of functional maturation of neurons. Therefore, we assessed the neurophysiological properties of iPSC-derived neurons when co-cultured with iSOX9-astrocytes. Until now, variable effects of PSC-derived astrocytes on neuronal maturation have been described via patch clamp [20, 22, 61, 62]. For our study, neurons generated by the inducible overexpression of *NGN2* from the *AAVS1* locus in PSCs [10, 12] were used. Those iNGN2-neurons on their own do not display electrical activity [49], however electrophysiological neuronal maturation is observed when co-cultured with PSC-derived astrocytes. Indeed, a recently submitted manuscript (Shih et al.) demonstrated that co-culture of DIV120 iPSC-derived astrocytes (generated via growth factors/small molecules) with iNGN2-neurons induced robust neuronal maturation. Here we demonstrated that the same iNGN2-neurons, when co-cultured with DIV40 iSOX9-astrocytes, acquire higher network activity measured by MEA analysis compared with rat neurons and iNGN2 co-cultures with growth factor/cytokine-induced DIV120 astrocytes. The ability of performing co-cultures with iSOX9-astrocytes already at DIV40 compared with the 120 days required in previous methods and their capacity to elicit optimal functional properties in human neurons, materializes a long standing objective of the stem cell field to create full human-based neural models for drug discovery research in neurosciences.

The creation of a stable iSOX9-PSC line to overcome the drawbacks associated with the use of lentiviral vectors is perhaps less suitable in a disease modeling setting, as creation of the line is time consuming. In such case, SOX9 overexpression via lentiviral transduction of NPCs following dual SMAD inhibition might be more practical. However, we have observed that in some patient-derived iPSCs, NPCs are more vulnerable to the stress caused by viral transduction. Therefore, the transgenic *AAVS1*-located iSOX9-astrocyte differentiation system will mainly have an impact on fundamental astrocyte research as well as screening/drug discovery purposes in the neuroscience field. In addition, it should be noted that all functional read-outs were performed using only one iPSC line, namely SIGi001-A. However, in the transcriptome analysis ALS-patient derived iSOX9-astrocytes were also included which clustered closely together with the SIGi001-A-derived iSOX9-astroyctes. Moreover, we also introduced the inducible *SOX9* coding sequence in the *AAVS1* locus of H9 embryonic stem cells (ESCs) and based on RT-qPCR and immunofluorescence, they could be similarly differentiated towards an astrocyte-like phenotype (Fig. S8).

In conclusion, we developed a robust, efficient and fast method to produce nearly 100 % pure functional astrocytes starting from PSCs by overexpression of *SOX9*, as the sole TF, in PSC-derived NPCs. Already on DIV35-40, iSOX9-astrocytes can be cryopreserved for downstream assays and phenocopy all functions of primary human astrocytes. Most importantly, iSOX9-astrocytes induce a higher degree of neuronal network formation in human PSC-derived neurons compared to gold-standard primary rat neuronal cultures. This high-yield protocol, requiring only ± 40 days of differentiation, should now enable creating high-throughput all-human iPSC-derived astrocyte-neuron co-cultures suitable to support drug discovery.

## Supplementary Information

ESM 1 Confirmation lentiviral overexpression of transcription factors via RT-qPCR. Gene expression measured via RT-qPCR of the transcription factors *NFI-A*, *NFI-B* and *SOX9* after their lentiviral overexpression, both alone or in combination (N=2-4; ^##^p<0.01 versus NFI-A, ^Δ^p<0.05 versus NFI-A+SOX9, ^+^p<0.05 versus all other conditions). All data represented as mean ± SEM (PDF 80 kb)

ESM 2 Lentiviral screen reveals that only *SOX9* overexpression in iPSC-derived NPCs upregulates astrocyte-specific markers. (A) Schematic overview: NPCs were generated from iPSCs via dual SMAD inhibition and afterwards transduced with lentiviral vectors (individually or different combinations) encoding for *NFI-A*, *NFI-B* and *SOX9*, controlled by a TET-ON promoter. Doxycycline was added for 7 consecutive days to induce overexpression. (B) Flow cytometry analysis of NPC progeny 7 days after adding doxycycline to asses the percentage of cells expressing S100B or EAAT1. (N=3; *p<0.05, **p<0.01). (C) RT-qPCR analysis of astrocytic, neuronal and pluripotency gene transcripts 7 days after lentiviral transduction and doxycycline addition (N=2-4; ^#^p<0.05 versus NFI-B). (D) Overview of RMCE to insert the *SOX9* CDS under a doxycycline inducible TET-ON promotor in the safe harbour *AAVS1* locus. (E) Flow cytometry analysis for GFP, before and after recombination of the *TET-ON-SOX9* cassette shows loss of GFP expression after RMCE (N=3; ****p<0.0001). (F) After RMCE, RT-qPCR for *SOX9* mRNA expression shows an upregulation following addition of doxycycline for three consecutive days (N=3; ****p<0,0001). All data represented as mean ± SEM. (PDF 164 kb)

ESM 3 Quality control of iPSC line after and before RMCE. (A) Junction PCR with one primer recognizing the *AAVS1* locus and one primer recognizing the GFP-Hygro-TK cassette flanked by FRT sites both at the 5 and 3 primed end of the cassette to confirm insertion in the *AAVS1* locus. (B) Southern Blot after digesting genomic DNA with NcoI and using a DIG-labeled probe recognizing the homology arm of the GFP-Hygro-TK cassette to exclude random integration. (C) After RMCE, in which the GFP-Hygro-TK cassette is exchanged for the *TET-ON-SOX9* cassette, no significant genome-wide aberrations were found via array-CGH. (D) Embryoid body (EB) Scorecard assay (https://www.thermofisher.com/be/en/home/life-science/stem-cell-research/taqman-hpsc-scorecard-panel/scorecard-software.html) implies pluripotency of SIGi001-A-20 iPSCs and differentiation potential towards the three lineages (ectoderm, mesoderm and endoderm). (PDF 465 kb)

ESM 4 Cryopreservation and thawing does not affect astrocytic phenotype of iSOX9-astrocytes. (A) Brightfield 5x image of DIV40 iSOX9-astrocytes after thawing. (B) Representative immunofluorescence images of thawed iSOX9-astrocytes which were frozen 22 days after stopping doxycycline (=DIV40) for S100B (green), GFAP (red), EAAT1 (green) and ALDH1L1 (red) (scale bar: 50 μm) (N=2 independent experiments). (PDF 4875 kb)

ESM 5 PCA of iSOX9-astrocytes, fHA, iCell astrocytes and published datasets. PCA plot, using all genes, of the included RNASeq samples with the transcriptomics data of Zhang *et al*., Li *et al.* and Tchieu *et al.* (PDF 47 kb)

ESM 6 Expression of “early” and “late” astrocyte marker genes in all included RNASeq samples. Gene expression of “early” (proliferation-related; *MKI67, TOP2A, TPX2, NUSAP1*) genes and “late” (astrocyte-related; *ALDH1L1, GFAP, EAAT1, AQP4*) genes in all included RNASeq samples. Results of One-Way ANOVA are only shown for the comparison with Zhang_fetal astrocytes (*p<0.05) and Zhang_postnatal astrocytes (#p<0.05) Data represented as mean ± SEM. (PDF 77 kb)

ESM 7 Gene ontology of development/age related astrocyte genes. Gene ontology analysis of all 11 clusters. The clusters contain genes of which the expression is at least 16-fold increased or decreased between the fetal and postnatal astrocytes from Zhang *et al*. (PDF 219 kb)

ESM 8 (PDF 87 kb)

ESM 9 Generation of iSOX9-H9 ESCs and differentiation towards iSOX9-astrocytes. (A) After RMCE to include the *TET-ON-SOX9* cassette in the *AAVS1* locus of H9 ESCs, RT-qPCR for *SOX9* mRNA expression demonstrated a robust upregulation following addition of doxycycline for three consecutive days (N=3; **p<0,01). Data represented as mean ± SEM. (B) RT-qPCR for *SOX9*, *S100B*, *GFAP*, *ALDH1L1* and *OCT4* transcripts at different time points throughout the differentiation (N=1 independent differentiation). (C) Representative immunofluorescence images of H9-derived iSOX9-astrocytes at 5 and 30 days after stopping doxycycline treatment for S100B (green), GFAP (red), EAAT1 (green) and ALDH1L1 (red) (scale bar: 50 μm). Quantification of the percentage of positive cells was performed using Columbus Image analysis software (PerkinElmer) (N=1 independent differentiation). (PDF 8137 kb)
