# Supplementary figures and images for: SOX9-induced Generation of Functional Astrocytes Supporting Neuronal Maturation in an All-human System
Source: Stem Cell Rev Rep. 2021 May 12;17(5):1855–73. doi: 10.1007/s12015-021-10179-x (PMC8553725; doi:10.1007/s12015-021-10179-x)

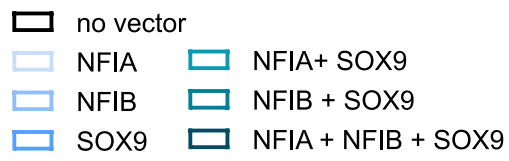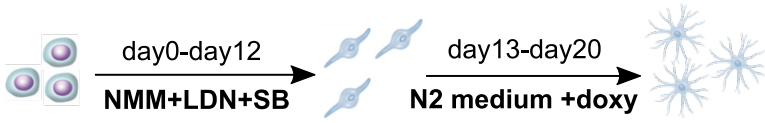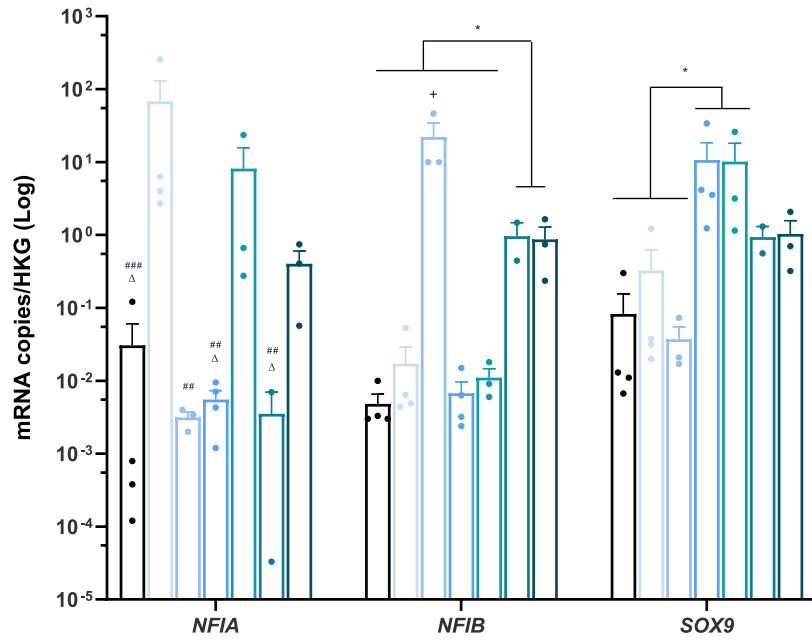

Supplement: Supplementary file 1 — Confirmation lentiviral overexpression of transcription factors via RT-qPCR. Gene expression measured via RT-qPCR of the transcription factors NFI-A, NFI-B and SOX9 after their lentiviral overexpression, both alone or in combination (N=2-4; ##p<0.01 versus NFI-A, Δp<0.05 versus NFI-A+SOX9, +p<0.05 versus all other conditions). All data represented as mean ± SEM (PDF 80 kb) [file 12015_2021_10179_MOESM1_ESM.pdf]

**A**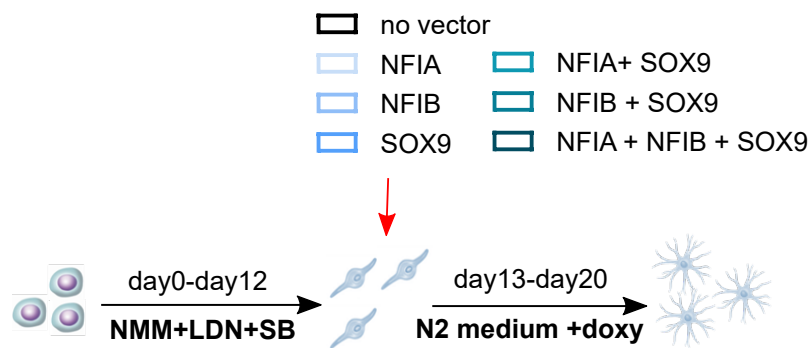**B**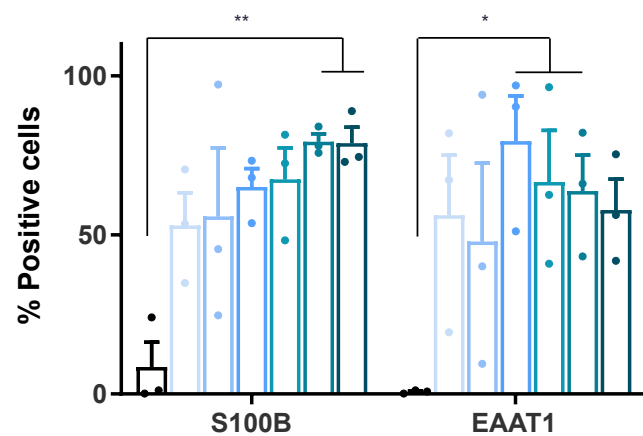**C**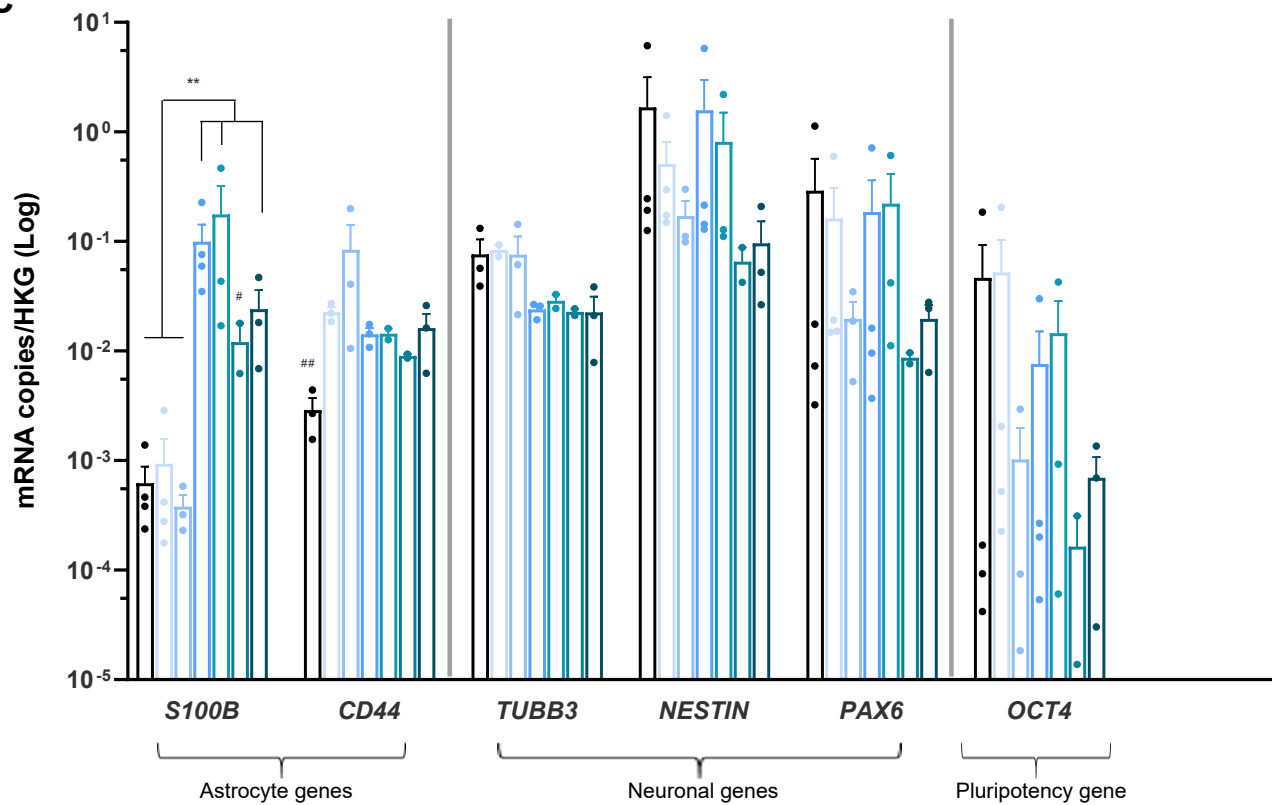**D**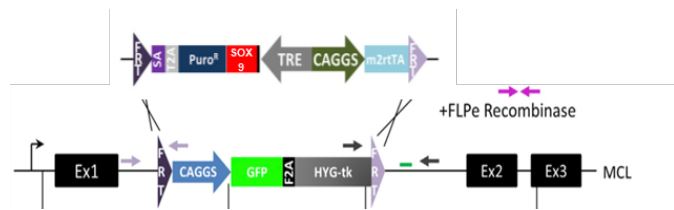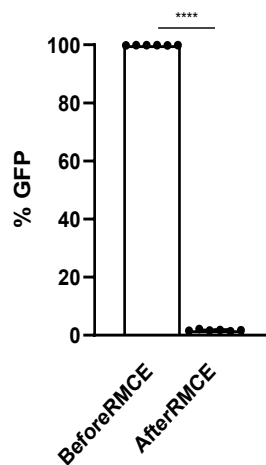**E**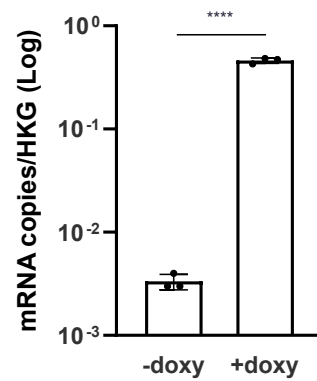

Supplement: Supplementary file 2 — Lentiviral screen reveals that only SOX9 overexpression in iPSC-derived NPCs upregulates astrocyte-specific markers. (A) Schematic overview: NPCs were generated from iPSCs via dual SMAD inhibition and afterwards transduced with lentiviral vectors (individually or different combinations) encoding for NFI-A, NFI-B and SOX9, controlled by a TET-ON promoter. Doxycycline was added for 7 consecutive days to induce overexpression. (B) Flow cytometry analysis of NPC progeny 7 days after adding doxycycline to asses the percentage of cells expressing S100B or EAAT1. (N=3; *p<0.05, **p<0.01). (C) RT-qPCR analysis of astrocytic, neuronal and pluripotency gene transcripts 7 days after lentiviral transduction and doxycycline addition (N=2-4; #p<0.05 versus NFI-B). (D) Overview of RMCE to insert the SOX9 CDS under a doxycycline inducible TET-ON promotor in the safe harbour AAVS1 locus. (E) Flow cytometry analysis for GFP, before and after recombination of the TET-ON-SOX9 cassette shows loss of GFP expression after RMCE (N=3; ****p<0.0001). (F) After RMCE, RT-qPCR for SOX9 mRNA expression shows an upregulation following addition of doxycycline for three consecutive days (N=3; ****p<0,0001). All data represented as mean ± SEM. (PDF 164 kb) [file 12015_2021_10179_MOESM2_ESM.pdf]

A

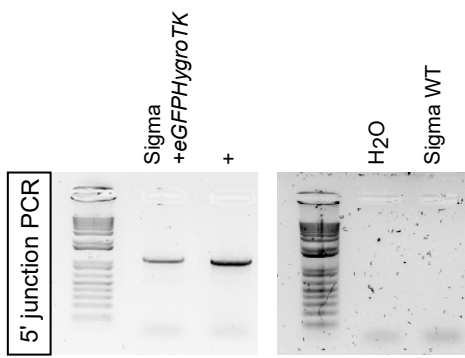

B

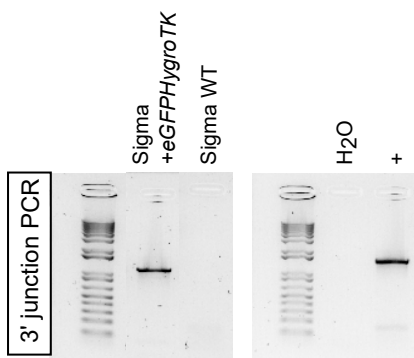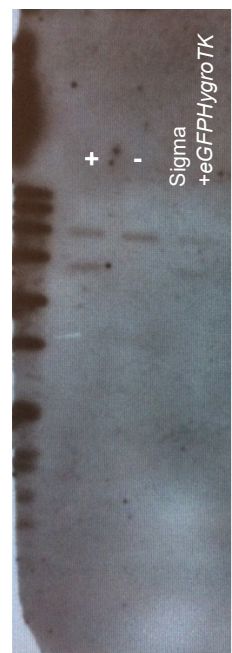

C

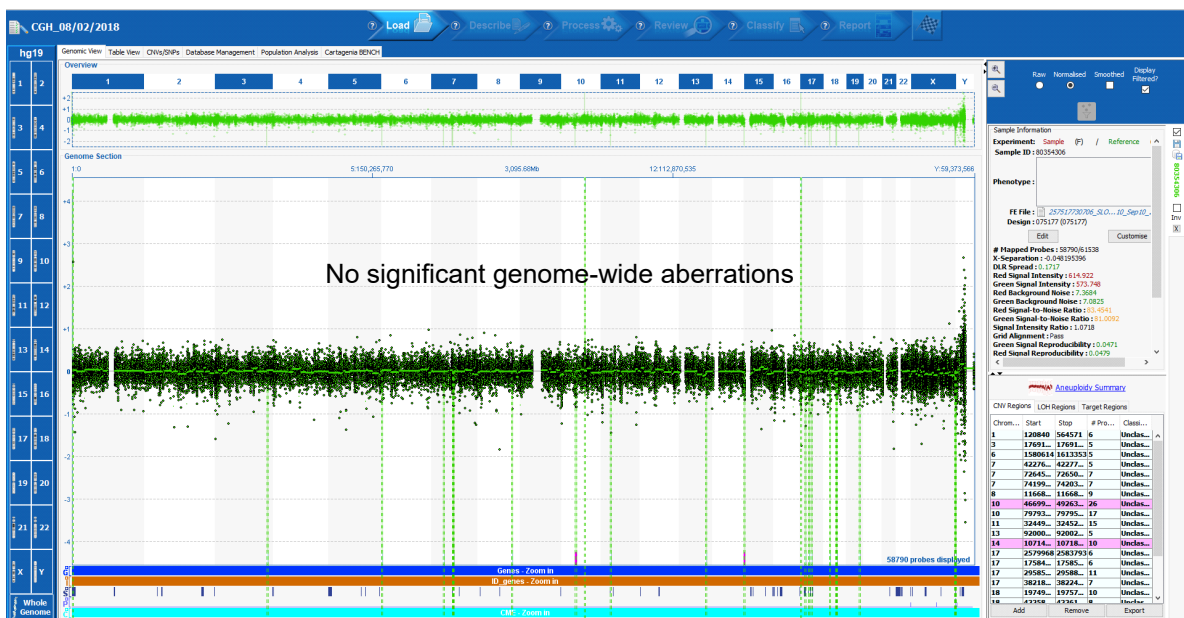

D

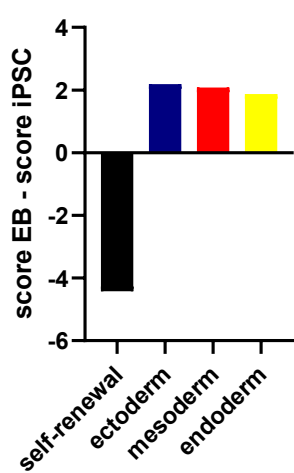

Supplement: Supplementary file 3 — Quality control of iPSC line after and before RMCE. (A) Junction PCR with one primer recognizing the AAVS1 locus and one primer recognizing the GFP-Hygro-TK cassette flanked by FRT sites both at the 5 and 3 primed end of the cassette to confirm insertion in the AAVS1 locus. (B) Southern Blot after digesting genomic DNA with NcoI and using a DIG-labeled probe recognizing the homology arm of the GFP-Hygro-TK cassette to exclude random integration. (C) After RMCE, in which the GFP-Hygro-TK cassette is exchanged for the TET-ON-SOX9 cassette, no significant genome-wide aberrations were found via array-CGH. (D) Embryoid body (EB) Scorecard assay (https://www.thermofisher.com/be/en/home/life-science/stem-cell-research/taqman-hpsc-scorecard-panel/scorecard-software.html) implies pluripotency of SIGi001-A-20 iPSCs and differentiation potential towards the three lineages (ectoderm, mesoderm and endoderm). (PDF 465 kb) [file 12015_2021_10179_MOESM3_ESM.pdf]

**A**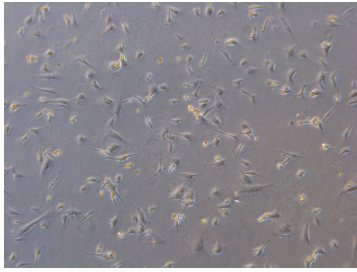**B**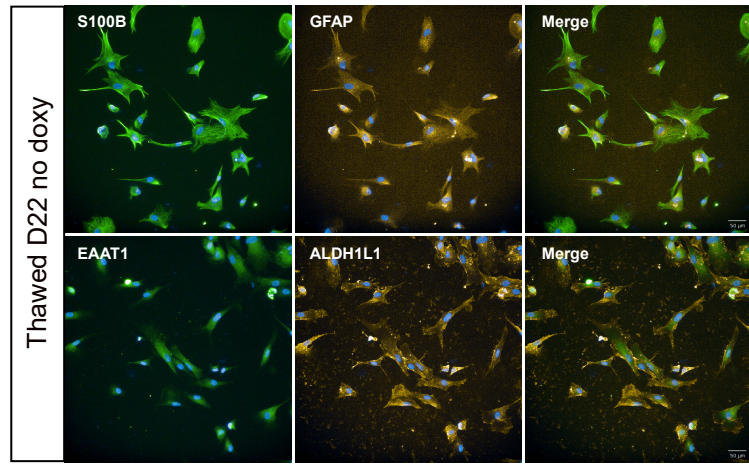

Supplement: Supplementary file 4 — Cryopreservation and thawing does not affect astrocytic phenotype of iSOX9-astrocytes. (A) Brightfield 5x image of DIV40 iSOX9-astrocytes after thawing. (B) Representative immunofluorescence images of thawed iSOX9-astrocytes which were frozen 22 days after stopping doxycycline (=DIV40) for S100B (green), GFAP (red), EAAT1 (green) and ALDH1L1 (red) (scale bar: 50 μm) (N=2 independent experiments). (PDF 4875 kb) [file 12015_2021_10179_MOESM4_ESM.pdf]

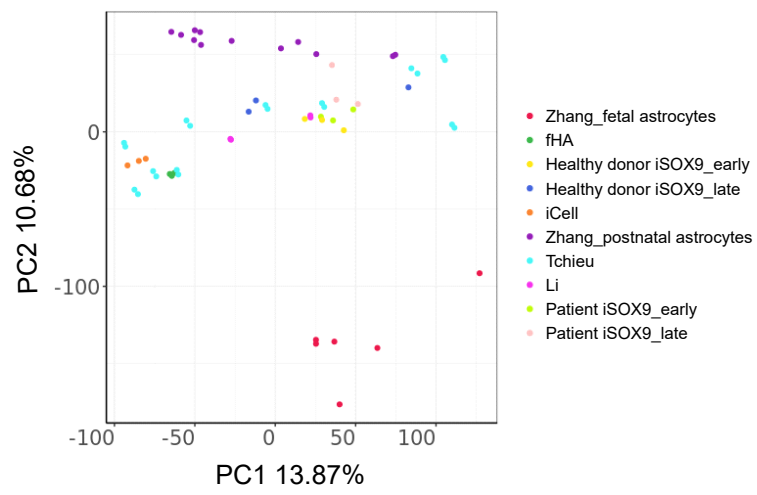

Supplement: Supplementary file 5 — PCA of iSOX9-astrocytes, fHA, iCell astrocytes and published datasets. PCA plot, using all genes, of the included RNASeq samples with the transcriptomics data of Zhang et al., Li et al. and Tchieu et al. (PDF 47 kb) [file 12015_2021_10179_MOESM5_ESM.pdf]

Biological process

Cellular component

Molecular function

Cluster 9

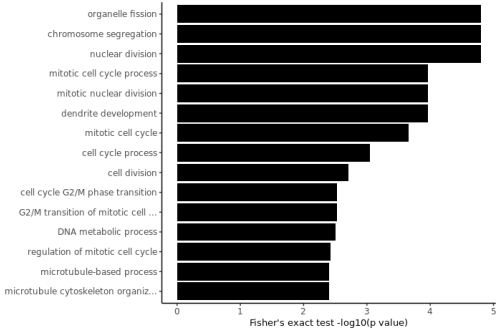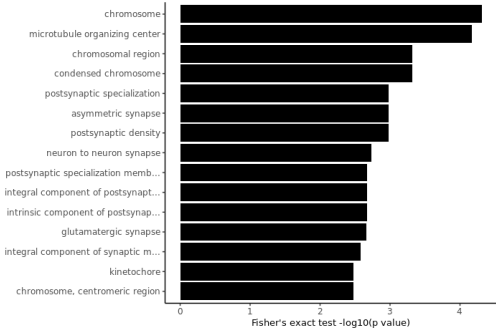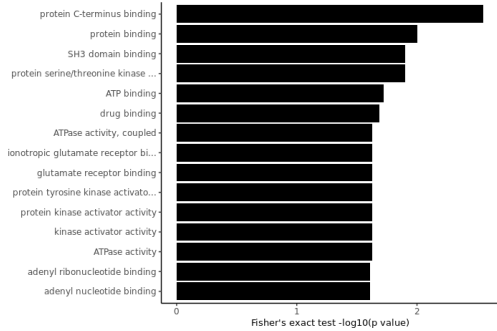

Cluster 11

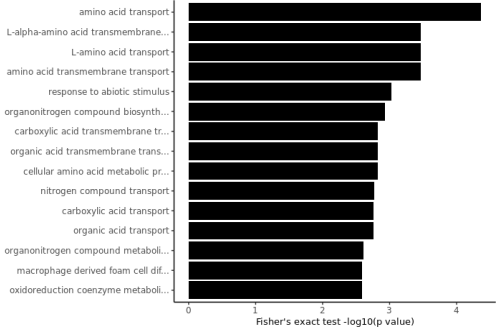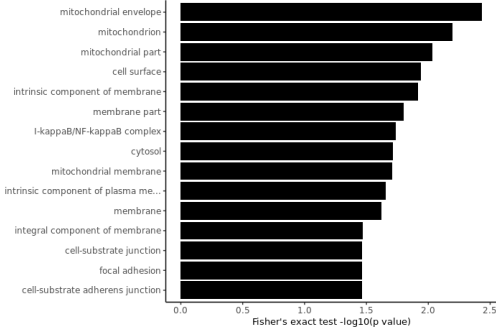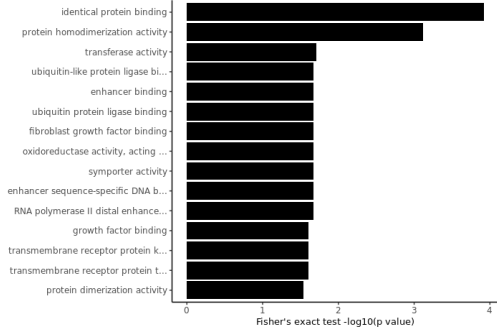

Supplement: Supplementary file 8 — (PDF 87 kb) [file 12015_2021_10179_MOESM8_ESM.pdf]

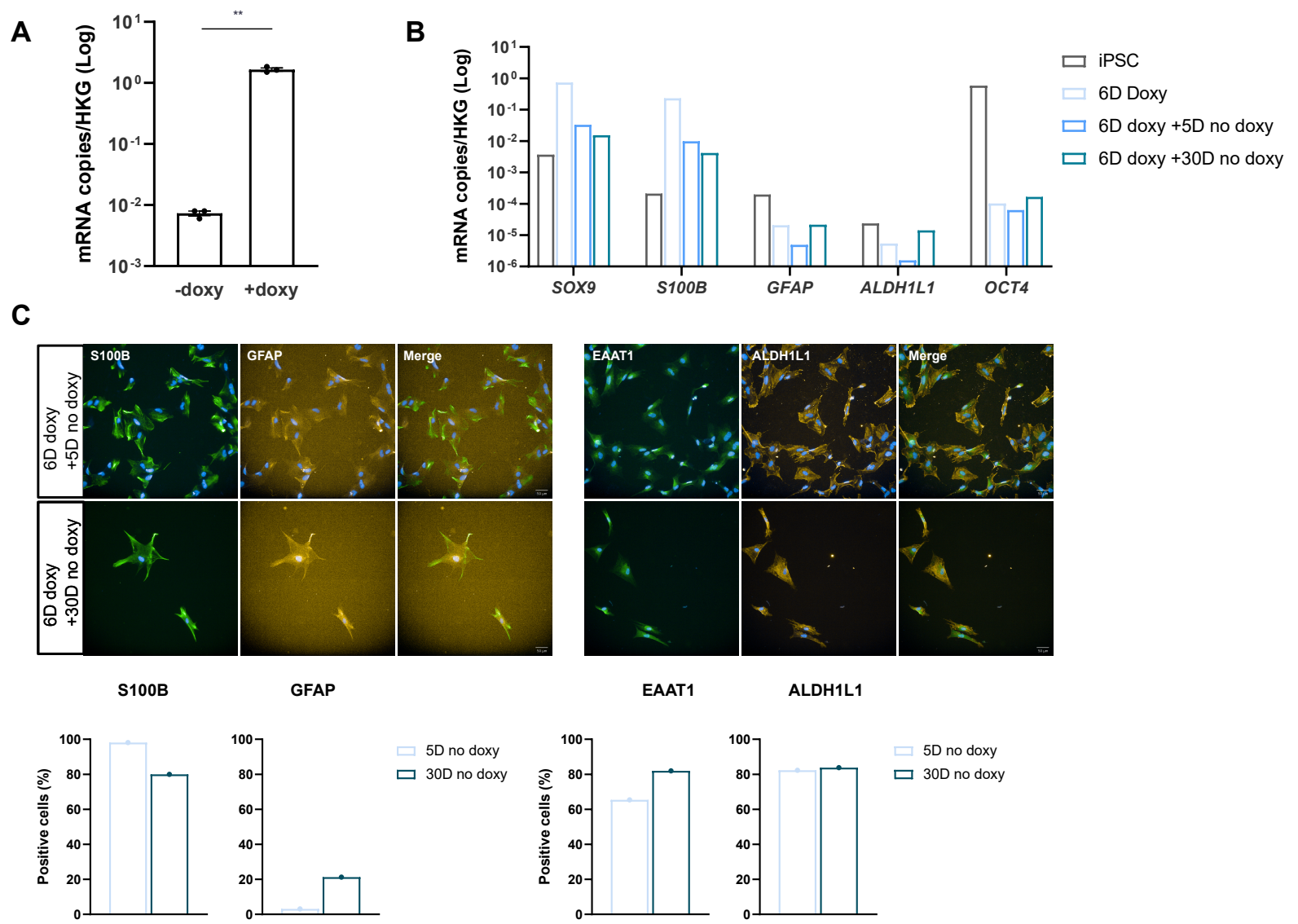

Supplement: Supplementary file 9 — Generation of iSOX9-H9 ESCs and differentiation towards iSOX9-astrocytes. (A) After RMCE to include the TET-ON-SOX9 cassette in the AAVS1 locus of H9 ESCs, RT-qPCR for SOX9 mRNA expression demonstrated a robust upregulation following addition of doxycycline for three consecutive days (N=3; **p<0,01). Data represented as mean ± SEM. (B) RT-qPCR for SOX9, S100B, GFAP, ALDH1L1 and OCT4 transcripts at different time points throughout the differentiation (N=1 independent differentiation). (C) Representative immunofluorescence images of H9-derived iSOX9-astrocytes at 5 and 30 days after stopping doxycycline treatment for S100B (green), GFAP (red), EAAT1 (green) and ALDH1L1 (red) (scale bar: 50 μm). Quantification of the percentage of positive cells was performed using Columbus Image analysis software (PerkinElmer) (N=1 independent differentiation). (PDF 8137 kb) [file 12015_2021_10179_MOESM9_ESM.pdf]
