# Supplementary material for: SOX9-induced Generation of Functional Astrocytes Supporting Neuronal Maturation in an All-human System
Source: Stem Cell Rev Rep. 2021 May 12;17(5):1855–73. doi: 10.1007/s12015-021-10179-x (PMC8553725; doi:10.1007/s12015-021-10179-x)
Supplement: Supplementary file 6 — Expression of “early” and “late” astrocyte marker genes in all included RNASeq samples. Gene expression of “early” (proliferation-related; MKI67, TOP2A, TPX2, NUSAP1) genes and “late” (astrocyte-related; ALDH1L1, GFAP, EAAT1, AQP4) genes in all included RNASeq samples. Results of One-Way ANOVA are only shown for the comparison with Zhang_fetal astrocytes (*p<0.05) and Zhang_postnatal astrocytes (#p<0.05) Data represented as mean ± SEM. (PDF 77 kb) [file 12015_2021_10179_MOESM6_ESM.pdf]

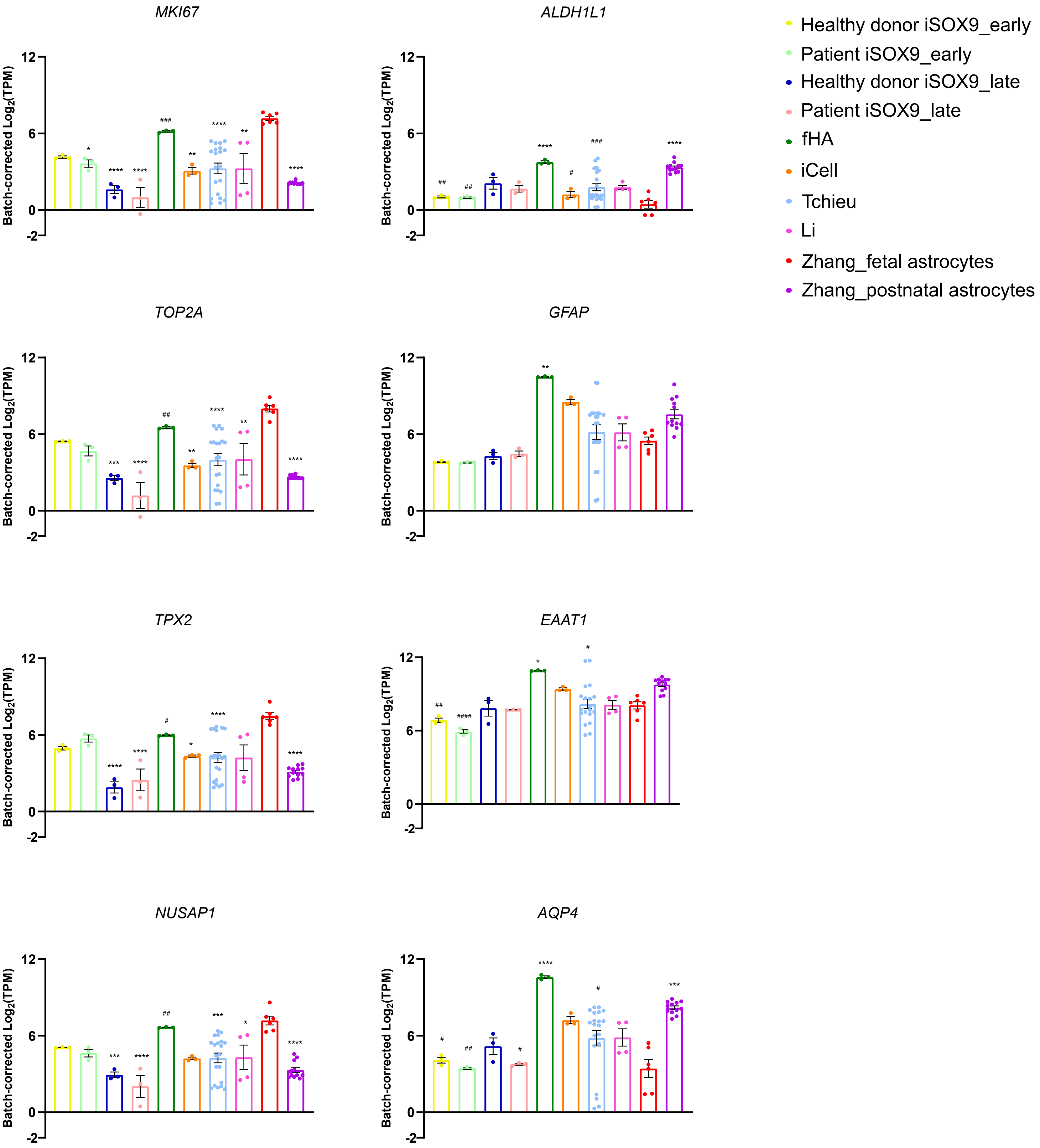
MKI67
ALDH1L1
TOP2A
GFAP
TPX2
EAAT1
NUSAP1
AQP4
Batch-corrected Log$_2$(TPM)
12
6
0
-2
Healthy donor iSOX9_early
Patient iSOX9_early
Healthy donor iSOX9_late
Patient iSOX9_late
fHA
iCell
Tchieu
Li
Zhang_fetal astrocytes
Zhang_postnatal astrocytes
