# Supplementary material for: SOX9-induced Generation of Functional Astrocytes Supporting Neuronal Maturation in an All-human System
Source: Stem Cell Rev Rep. 2021 May 12;17(5):1855–73. doi: 10.1007/s12015-021-10179-x (PMC8553725; doi:10.1007/s12015-021-10179-x)
Supplement: Supplementary file 7 — Gene ontology of development/age related astrocyte genes. Gene ontology analysis of all 11 clusters. The clusters contain genes of which the expression is at least 16-fold increased or decreased between the fetal and postnatal astrocytes from Zhang et al. (PDF 219 kb) [file 12015_2021_10179_MOESM7_ESM.pdf]

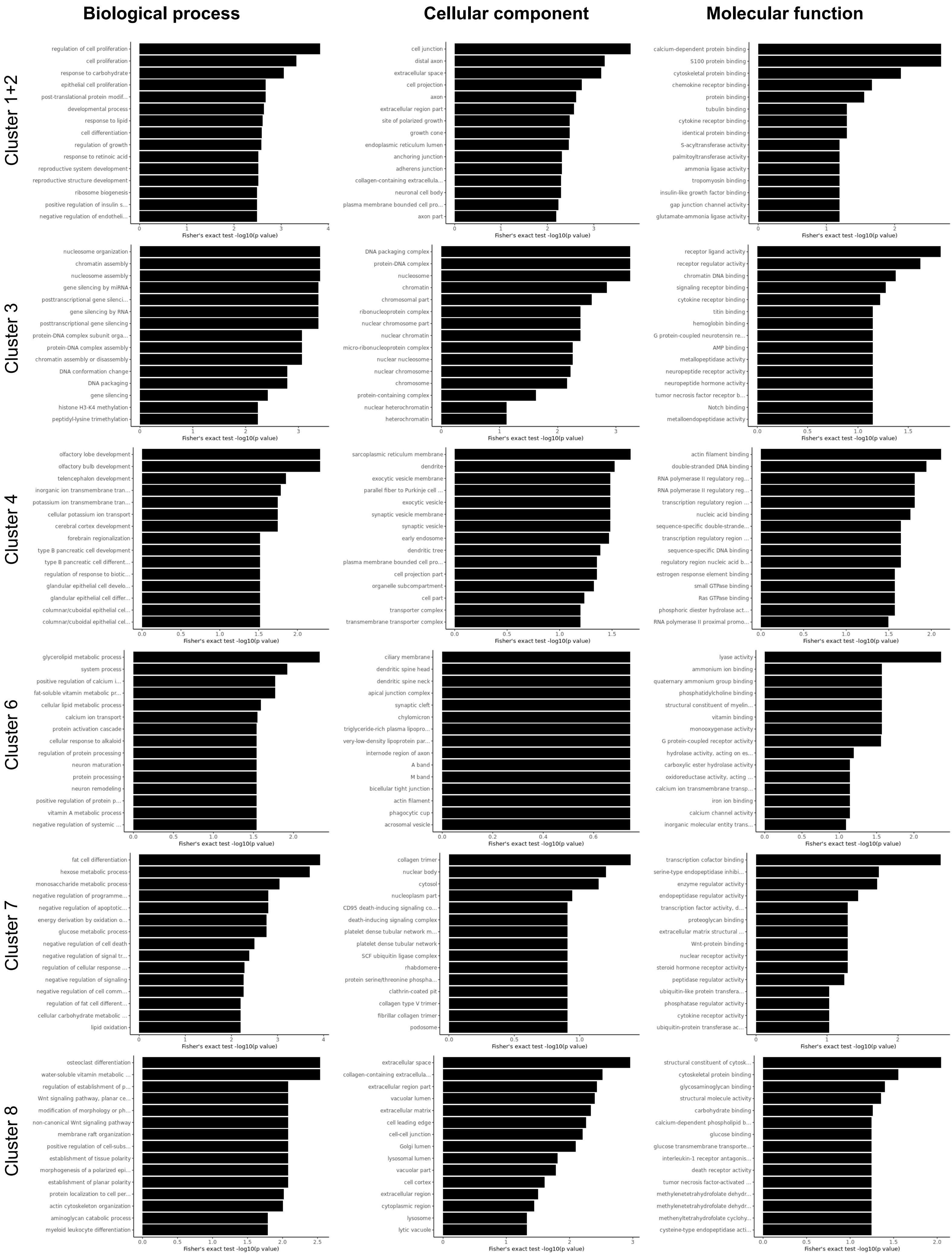
Biological process
Cellular component
Molecular function
Cluster 1+2
regulation of cell proliferation
cell proliferation
response to carbohydrate
epithelial cell proliferation
post-translational protein modif...
developmental process
response to lipid
cell differentiation
regulation of growth
response to retinoic acid
reproductive system development
reproductive structure development
ribosome biogenesis
positive regulation of insulin s...
negative regulation of endotheli...
Fisher's exact test -log10(p value)
cell junction
distal axon
extracellular space
cell projection
axon
extracellular region part
site of polarized growth
growth cone
endoplasmic reticulum lumen
anchoring junction
adherens junction
collagen-containing extracellula...
neuronal cell body
plasma membrane bounded cell pro...
axon part
Fisher's exact test -log10(p value)
calcium-dependent protein binding
S100 protein binding
cytoskeletal protein binding
chemokine receptor binding
protein binding
tubulin binding
cytokine receptor binding
identical protein binding
S-acyltransferase activity
palmitoyltransferase activity
ammonia ligase activity
tropomyosin binding
insulin-like growth factor binding
gap junction channel activity
glutamate-ammonia ligase activity
Fisher's exact test -log10(p value)
Cluster 3
nucleosome organization
chromatin assembly
nucleosome assembly
gene silencing by miRNA
posttranscriptional gene silenci...
gene silencing by RNA
posttranscriptional gene silencing
protein-DNA complex subunit orga...
protein-DNA complex assembly
chromatin assembly or disassembly
DNA conformation change
DNA packaging
gene silencing
histone H3-K4 methylation
peptidyl-lysine trimethylation
Fisher's exact test -log10(p value)
DNA packaging complex
protein-DNA complex
nucleosome
chromatin
chromosomal part
ribonucleoprotein complex
nuclear chromosome part
nuclear chromatin
micro-ribonucleoprotein complex
nuclear nucleosome
nuclear chromosome
chromosome
protein-containing complex
nuclear heterochromatin
heterochromatin
Fisher's exact test -log10(p value)
receptor ligand activity
receptor regulator activity
chromatin DNA binding
signaling receptor binding
cytokine receptor binding
titin binding
hemoglobin binding
G protein-coupled neurotensin re...
AMP binding
metallopeptidase activity
neuropeptide receptor activity
neuropeptide hormone activity
tumor necrosis factor receptor b...
Notch binding
metalloendopeptidase activity
Fisher's exact test -log10(p value)
Cluster 4
olfactory lobe development
olfactory bulb development
telencephalon development
inorganic ion transmembrane tran...
potassium ion transmembrane tran...
cellular potassium ion transport
cerebral cortex development
forebrain regionalization
type B pancreatic cell development
type B pancreatic cell different...
regulation of response to biotic...
glandular epithelial cell develo...
glandular epithelial cell differ...
columnar/cuboidal epithelial cel...
columnar/cuboidal epithelial cel...
Fisher's exact test -log10(p value)
sarcoplasmic reticulum membrane
dendrite
exocytic vesicle membrane
parallel fiber to Purkinje cell ...
exocytic vesicle
synaptic vesicle membrane
synaptic vesicle
early endosome
dendritic tree
plasma membrane bounded cell pro...
cell projection part
organelle subcompartment
cell part
transporter complex
transmembrane transporter complex
Fisher's exact test -log10(p value)
actin filament binding
double-stranded DNA binding
RNA polymerase II regulatory reg...
RNA polymerase II regulatory reg...
transcription regulatory region ...
nucleic acid binding
sequence-specific double-strande...
transcription regulatory region ...
sequence-specific DNA binding
regulatory region nucleic acid b...
estrogen response element binding
small GTPase binding
Ras GTPase binding
phosphoric diester hydrolase act...
RNA polymerase II proximal promo...
Fisher's exact test -log10(p value)
Cluster 6
glycerolipid metabolic process
system process
positive regulation of calcium i...
fat-soluble vitamin metabolic pr...
cellular lipid metabolic process
calcium ion transport
protein activation cascade
cellular response to alkaloid
regulation of protein processing
neuron maturation
protein processing
neuron remodeling
positive regulation of protein p...
vitamin A metabolic process
negative regulation of systemic ...
Fisher's exact test -log10(p value)
ciliary membrane
dendritic spine head
dendritic spine neck
apical junction complex
synaptic cleft
chylomicron
triglyceride-rich plasma lipopro...
very-low-density lipoprotein par...
internode region of axon
A band
M band
bicellular tight junction
actin filament
phagocytic cup
acrosomal vesicle
Fisher's exact test -log10(p value)
lyase activity
ammonium ion binding
quaternary ammonium group binding
phosphatidylcholine binding
structural constituent of myelin...
vitamin binding
monooxygenase activity
G protein-coupled receptor activity
hydrolase activity, acting on es...
carboxylic ester hydrolase activity
oxidoreductase activity, acting ...
calcium ion transmembrane transp...
iron ion binding
calcium channel activity
inorganic molecular entity trans...
Fisher's exact test -log10(p value)
Cluster 7
fat cell differentiation
hexose metabolic process
monosaccharide metabolic process
negative regulation of programme...
negative regulation of apoptotic...
energy derivation by oxidation o...
glucose metabolic process
negative regulation of cell death
negative regulation of signal tr...
regulation of cellular response ...
negative regulation of signaling
negative regulation of cell comm...
regulation of fat cell different...
cellular carbohydrate metabolic ...
lipid oxidation
Fisher's exact test -log10(p value)
collagen trimer
nuclear body
cytosol
nucleoplasm part
CD95 death-inducing signaling co...
death-inducing signaling complex
platelet dense tubular network m...
platelet dense tubular network
SCF ubiquitin ligase complex
rhabdomere
protein serine/threonine phospha...
clathrin-coated pit
collagen type V trimer
fibrillar collagen trimer
podosome
Fisher's exact test -log10(p value)
transcription cofactor binding
serine-type endopeptidase inhibi...
enzyme regulator activity
endopeptidase regulator activity
transcription factor activity, d...
proteoglycan binding
extracellular matrix structural ...
Wnt-protein binding
nuclear receptor activity
steroid hormone receptor activity
peptidase regulator activity
ubiquitin-like protein transfera...
phosphatase regulator activity
cytokine receptor activity
ubiquitin-protein transferase ac...
Fisher's exact test -log10(p value)
Cluster 8
osteoclast differentiation
water-soluble vitamin metabolic ...
regulation of establishment of p...
Wnt signaling pathway, planar ce...
modification of morphology or ph...
non-canonical Wnt signaling pathway
membrane raft organization
positive regulation of cell-subs...
establishment of tissue polarity
morphogenesis of a polarized epi...
establishment of planar polarity
protein localization to cell per...
actin cytoskeleton organization
aminoglycan catabolic process
myeloid leukocyte differentiation
Fisher's exact test -log10(p value)
extracellular space
collagen-containing extracellula...
extracellular region part
vacuolar lumen
extracellular matrix
cell leading edge
cell-cell junction
Golgi lumen
lysosomal lumen
vacuolar part
cell cortex
extracellular region
cytoplasmic region
lysosome
lytic vacuole
Fisher's exact test -log10(p value)
structural constituent of cytosk...
cytoskeletal protein binding
glycosaminoglycan binding
structural molecule activity
carbohydrate binding
calcium-dependent phospholipid b...
glucose binding
glucose transmembrane transporte...
interleukin-1 receptor antagonis...
death receptor activity
tumor necrosis factor-activated ...
methylenetetrahydrofolate dehydr...
methylenetetrahydrofolate dehydr...
methenyltetrahydrofolate cyclohy...
cysteine-type endopeptidase acti...
Fisher's exact test -log10(p value)
